# Supplementary material for: Biosensor Architectures for High-Fidelity Reporting of Cellular Signaling
Source: Biophys J. 2014 Aug 5;107(3):773–82. doi: 10.1016/j.bpj.2014.06.021 (PMC4129486; doi:10.1016/j.bpj.2014.06.021)
Supplement: Document S1. Nondimensional analysis, approximate analytical solutions, BioNetGen code used to generate the full model, and seven figures [file mmc1.pdf]

Supporting Materials:
Biosensor architectures for high fidelity reporting of cellular signaling

Omer Dushek[1,2,¶], Annemarie C Lellouch[3,4,5], David J. Vaux[1], Vahid Shahrezaei[6,¶]

[1]Sir William Dunn School of Pathology, University of Oxford, UK
[2]Wolfson Centre for Mathematical Biology, Mathematical Institute, University of Oxford, UK
[3]Aix Marseille Université, Laboratoire d'Adhésion et Inflammation, Marseille, 13288, France
[4]Inserm U1067, Marseille, 13288, France
[5]CNRS UMR 7333, Marseille, 13288, France
[6]Department of Mathematics, Imperial College London, UK
[¶]Corresponding authors

Non-dimensional analysis of a reduced model

We consider a reduced model that includes both intramolecular and intermolecular reactions but introduce the simplification that the maximum oligomer that can be formed is a dimer. In this reduced model all possible reactions can be enumerated as follows,

$$E + B \underset{k^e_{\text{off}}}{\overset{k^e_{\text{on}}}{\rightleftharpoons}} C \overset{k^e_{\text{cat}}}{\rightharpoonup} E + B^*$$

$$F + B^* \underset{k^f_{\text{off}}}{\overset{k^f_{\text{on}}}{\rightleftharpoons}} D \overset{k^f_{\text{cat}}}{\rightharpoonup} F + B$$

$$B^* \underset{k^1_{\text{off}}}{\overset{k^1_{\text{on}}}{\rightleftharpoons}} B^*_C, \quad B^* + B^* \underset{k^2_{\text{off}}}{\overset{k^2_{\text{on}}}{\rightleftharpoons}} B^*_2, \quad B^* + B \underset{k^2_{\text{off}}}{\overset{k^2_{\text{on}}}{\rightleftharpoons}} B_2$$

The corresponding non-dimensional steady-state equations are:

$$\lambda_1 \widehat{C} = \widehat{B}\widehat{E}$$
$$\lambda_2 \widehat{D} = \widehat{B}^* \widehat{F}$$
$$\lambda_3 \widehat{B}^*_C = \widehat{B}^*$$
$$\lambda_4 \widehat{B}^*_2 = 2(\widehat{B}^*)^2$$
$$\lambda_4 \widehat{B}_2 = 2\widehat{B}^* \widehat{B}$$
$$\lambda_5 \lambda_7 \widehat{C} = \widehat{D}$$
$$1 = \widehat{E} + \widehat{C}$$
$$1 = \widehat{F} + \widehat{D}$$
$$1 = \widehat{B} + \widehat{B}^* + \lambda_6 \lambda_7 \widehat{C} + \lambda_6 \widehat{D} + \widehat{B}^*_C + (\widehat{B}^*_2 + \widehat{B}_2)$$

where 'hat' quantities are the non-dimensional concentrations and the λ_i are the non-dimensional parameters defined as follows: $\widehat{E} = E/E_T$, $\widehat{F} = F/F_T$, $\widehat{C} = C/E_T$, $\widehat{D} = D/F_T$, $\widehat{B} = B/B_T$, $\widehat{B^*} = B^*/B_T$, $\widehat{B^*_C} = B^*_C/B_T$, $\widehat{B_2} = B_2/(B_T/2)$,$\widehat{B^*_2} = B^*_2/(B_T/2)$,$\lambda_1 = K^{\mathrm{e}}_{\mathrm{M}}/B_T$, $\lambda_2 = K^{f}_{\mathrm{M}}/B_T$, $\lambda_3 = K^1_{\mathrm{D}}$, $\lambda_4 = K^2_{\mathrm{D}}/B_T$, $\lambda_5 = k^{\mathrm{e}}_{\mathrm{cat}}/k^{f}_{\mathrm{cat}}$, $\lambda_6 = F_T/B_T$, and $\lambda_7 = E_T/F_T$. We make the simplification that the binding and catalytic activities are identical for the two enzymes ($\lambda_1 = \lambda_2$, $\lambda_5 = 1$) which reduces the number of non-dimensional parameters to 5.

We generate biosensor response curves by solving the system of equations using *fsolve* in Matlab (Mathework, MA) for increasing values of λ_7 whilst fixing the values of λ_1, λ_3, λ_4, and λ_6. We determine the dynamic range, potency, sensitivity, and the amount of free signaling protein (E) at the EC_{50} for each resulting biosensor response curve (as described above) and repeat the calculation for different values of the non-dimensional parameters to generate the heat maps (Fig. S7). In the case of the intramolecular biosensor (Fig. S7A-D) we fix $\lambda_4 \gg 1$ while varying λ_1 and λ_3. In the case of the intermolecular biosensor (Fig. S7E-H) we fix $\lambda_3 \gg 1$ while varying λ_1 and λ_4. In both cases we fix $\lambda_6 = 0.01$ to allow enzyme saturation and ultrasensitivity; if $\lambda_6 = 1$ then ultrasensitivity is never observed. Using these heat maps we determine the high fidelity reporting region (Fig. 4).

Approximate analytical solution

Under quasi-steady state assumption, kinase activity (v_E) and phosphatase activity (v_F) can be described using Michaelis-Menten kinetics,

$$v_E = \frac{dB^*}{dt} = \frac{k^e_{\mathrm{cat}} E B}{K^e_M + B} \tag{1}$$

$$v_F = \frac{dB}{dt} = \frac{k^f_{\mathrm{cat}} F B^*}{K^f_M + B^*} \tag{2}$$

where, K^e_M and K^f_M are Michaelis-constants for the kinase and phosphatase, respectively. Allowing intermolecular and intramolecular biosensor interactions and limiting ourselves to the case where maximum oligomer is a dimer, we have the following conservation equation for the total biosensor (B_T),

$$B_T = B + B^* + B^*_c + 2B_2 + 2B^*_2 \tag{3}$$

Assuming that intermolecular and intramolecular reactions take place faster than enzymatic reactions, we can use a fast equilibrium approximation to obtain expressions for B_c^*, B_2 and B_2^* in terms of free B and B^*. Substituting these in the equation for B_T, we have,

$$B_T = B + B^* + B^*/K_D^1 + 2BB^*/K_D^2 + 2B^*B^*/K_D^2 \quad (4)$$

where, K_D^1 and K_D^2 are the dissociation constants of the intramolecular and intermolecular interactions, respectively. By solving for B in Eqn. 4, we obtain,

$$B = \frac{B_T - (1 + 1/K_D^1)B^* - 2B^{*2}/K_D^2}{1 + 2B^*/K_D^2} \quad (5)$$

We can substitute B as a function of B^* in Eqn. 1 above. The kinase and phosphtase activity then can be plotted as a function of B^* and their intersection represent the equilibrium value of B^*. An analytical expression for B^* as a root of the cubic equation can be obtained by equating Eqn. 1 and 2 or alternatively, the equilibrium B^* can be obtained numerically as we have done in Fig. S5.

This analysis is also helpful to shed light on the dependence of EC_{50} to model parameters. At $E = EC_{50}$, we denote concentration of B and B^* by B_{EC50} and B^*_{EC50}. By equating v_E and v_F from Eqn. 1 and 2 at EC_{50} we obtain:

$$EC_{50} = F \frac{k_{\text{cat}}^f}{k_{\text{cat}}^e} \frac{B^*_{EC50}}{B_{EC50}} \frac{K_M^e + B_{EC50}}{K_M^f + B^*_{EC50}} \quad (6)$$

In the presence of intermolecular or intramolecular interactions and due to sequestration of free biosensor maximum B^* is smaller than B_T (can be seen from Eqn. 4 for $B = 0$) while the maximum free B is still B_T. Similarly, it can be seen that at $E = EC_{50}$, we also have $B^*_{EC50} < B_{EC50}$. As can be seen from Eqn. 6 this results in always a reduction in EC_{50}. Also, if K_Ms are small this effect become weaker as EC_50 becomes independent of B_{EC50} and B^*_{EC50}. These match the results shown in Fig. S7.

This analysis shows that the mechanism of reduced ultrasensitivity is substrate sequestration. For the sequestration to work, we have assumed throughout this paper that B and B^* cannot be modified while in the B_c^*, B_2 and B_2^* states (or in higher order oligomeric states in the general model). Assuming that B^* cannot be dephosphorylated in B_2 is critical to our results but we can relax this assumption about B within the B_2 complex. In this case, we need to replace Eqn. 1 by

$$v_E \quad = \frac{dB^*}{dt} \quad = \frac{k_{\text{cat}}^e E(B + B^2)}{K_M^e + B + B^2} \quad (7)$$

So, we can solve for $B + B^2$ from Eqn. 4 and substitute it into Eqn. 7 and repeat the analysis in Fig. S5. This analysis suggests that assuming that B cannot be modified while in the B^2 state contributes to the decrease in ultrasensitivity and contributes to maintaining a constant EC_{50} but does not change our qualitative conclusions (not shown).

BioNetGen code used to generate mathematical model

```
#Model of a kinase (E) and phosphatase (F) actin on a biosensor (B).

begin parameters

#Modification module

#Enzymatic reaction (kinase)
Ekf     10
Ekb     1
Ekc     1

#Enzymatic reaction (phosphatase)
Fkf     10
Fkb     1
Fkc     1

#Intramolecular module
kon1    1000
koff1   1

#Intermolecular module
kon2    10
koff2   1

end parameters

begin molecule types
E(b)
F(b)
B(e~0~1,b,Y~U~P)
end  molecule types

begin seed species
E(b)            1
F(b)            1
B(e~0,b,Y~U)    100
end seed species

begin reaction rules

#Phosphorylation by E.
E(b) + B(e~0,b,Y~U) <-> E(b!1).B(e~1,b,Y~U!1)   Ekf,Ekb
E(b!1).B(e~1,b,Y~U!1) -> E(b) + B(e~0,b,Y~P)    Ekc

#Dephosphorylation by F.
F(b) + B(e~0,b,Y~P) <-> F(b!1).B(e~1,b,Y~P!1)   Fkf,Fkb
F(b!1).B(e~1,b,Y~P!1) -> F(b) + B(e~0,b,Y~U)    Fkc

#Intramolecular reaction.
B(e~0,b,Y~P) <-> B(e~0,b!1,Y~P!1)    kon1,koff1

#Intermolecular reaction.
```

```
B(e~0,Y~P) + B(e~0,b) <-> B(e~0,Y~P!1).B(e~0,b!1)    kon2,koff2

end reaction rules

begin observables
#Biosensor in State 1
    Molecules W B(e~0,b,Y~P),B(e~0,b,Y~U),B(e~1,b)
#Biosensor in State 2
    Molecules U B(e~0,b!1,Y~P!1)
#Biosensor in State 3 (combination of all states below, see manuscript for
   details)
    Species   V2 B==2
    Species   V3 B==3
    Species   V4 B==4
    Species   V5 B==5
    Species   V6 B==6
    Species   V7 B==7
    Species   V8 B==8
    Species   V9 B==9
    Species   V10 B==10
    Species   V11 B==11
    Species   V12 B==12
    Species   V13 B==13
    Species   V14 B==14
    Species   V15 B==15
#Sequestered signaling protein
    Molecules  E E(b!+)
end observables

begin actions

#Generate network with a maximum oligomer of size 15
generate_network({overwrite=>1,max_agg=>15});

#Generate network with a maximum oligomer of size 2
generate_network({overwrite=>1,max_agg=>2});

#Generate Matlab file
writeMfile({});

end actions
```

Supporting Figures

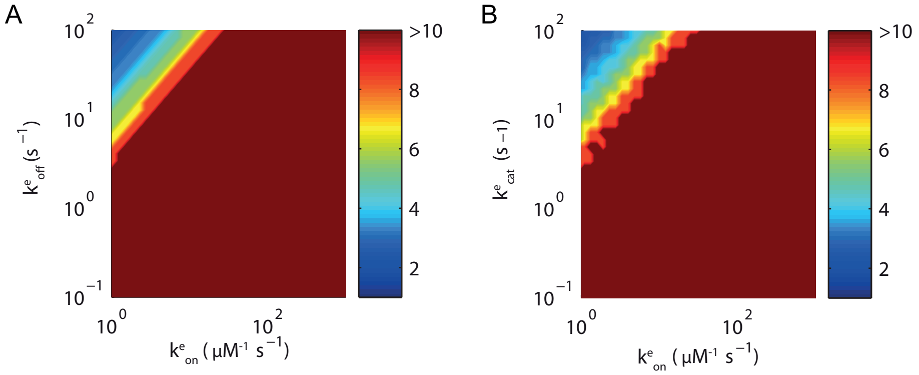

Fig S1: Effects of modifying the kinase-biosensor binding and reaction parameters on the Hill number for the biosensor architecture in Fig. 2. Heat maps of the Hill number determined from biosensor response curves as a function of the kinase-biosensor A) on-rate and off-rate and B) on-rate and catalytic-rate. Reductions in Hill numbers are observed when the off-rate or catalytic-rate are large and when the on-rate is small because in this regime, the Michaelis-constant (K_M) is large. Parameters: [Biosensor]= $B_{\mathrm{T}} = 100$ μM, $F_{\mathrm{T}} = 1$ μM, $k^1_{\mathrm{on}} = 10$ s^{-1}, $k^1_{\mathrm{off}} = 1$ s^{-1}, $k^e_{\mathrm{on}} = k^f_{\mathrm{on}}$ =10 μM^{-1}s^{-1}, $k^e_{\mathrm{off}} = k^e_{\mathrm{off}} = 1$ s^{-1}, $k^e_{\mathrm{cat}} = k^f_{\mathrm{cat}} = 1$ s^{-1}.

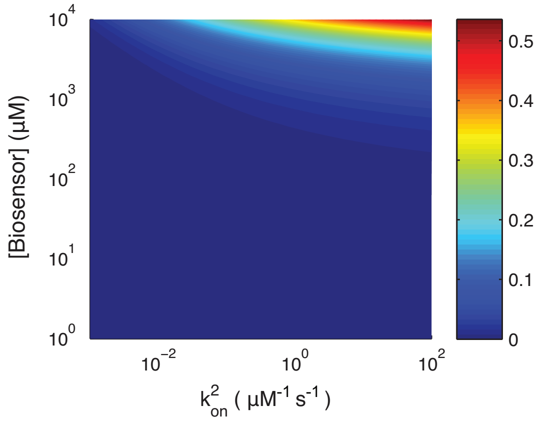

Fig S2: Heat map showing the fraction of biosensor in an oligomer of size 15 (maximum oligomer size) as a function of the intermolecular on-rate (x-axis) and the biosensor concentration (y-axis) for the parameters used to generate Fig. 3. The fraction is found to be less than 1% over the majority of parameter space indicating that approximating the maximum number of biosensors in an oligomer to 15 does not introduce appreciable errors.

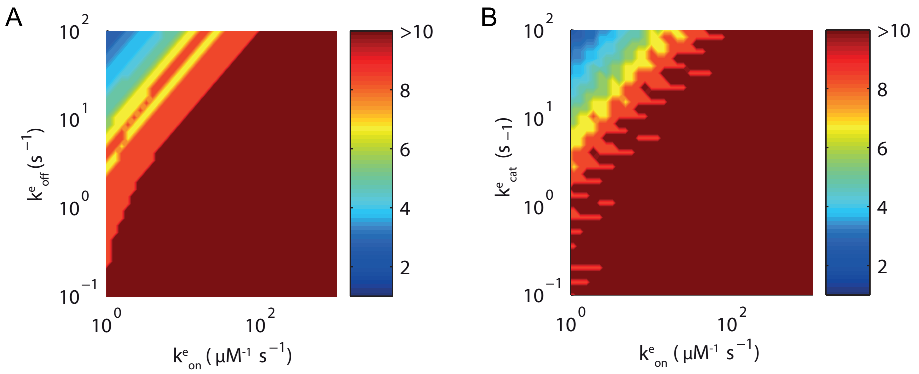

Fig S3: Effects of modifying the kinase-biosensor binding and reaction parameters on the Hill number for the biosensor architecture in Fig. 3. Heat maps of the Hill number determined from biosensor response curves as a function of the kinase-biosensor A) on-rate and off-rate and B) on-rate and catalytic-rate. As in Fig. S1, reductions in Hill numbers are observed when the Michaelis-constant (K_M) is large. Parameters: [Biosensor]= $B_{\text{T}} = 100\ \mu$M, $F_{\text{T}} = 1\ \mu$M, $k^{2}_{\text{on}} = 0.1\ \mu\text{M}^{-1}\text{s}^{-1}$, $k^{2}_{\text{off}} = 1\ \text{s}^{-1}$, $k^{e}_{\text{on}} = k^{f}_{\text{on}}$ =10 $\mu\text{M}^{-1}\text{s}^{-1}$, $k^{e}_{\text{off}} = k^{e}_{\text{off}} = 1\ \text{s}^{-1}$, $k^{e}_{\text{cat}} = k^{f}_{\text{cat}} = 1\ \text{s}^{-1}$.

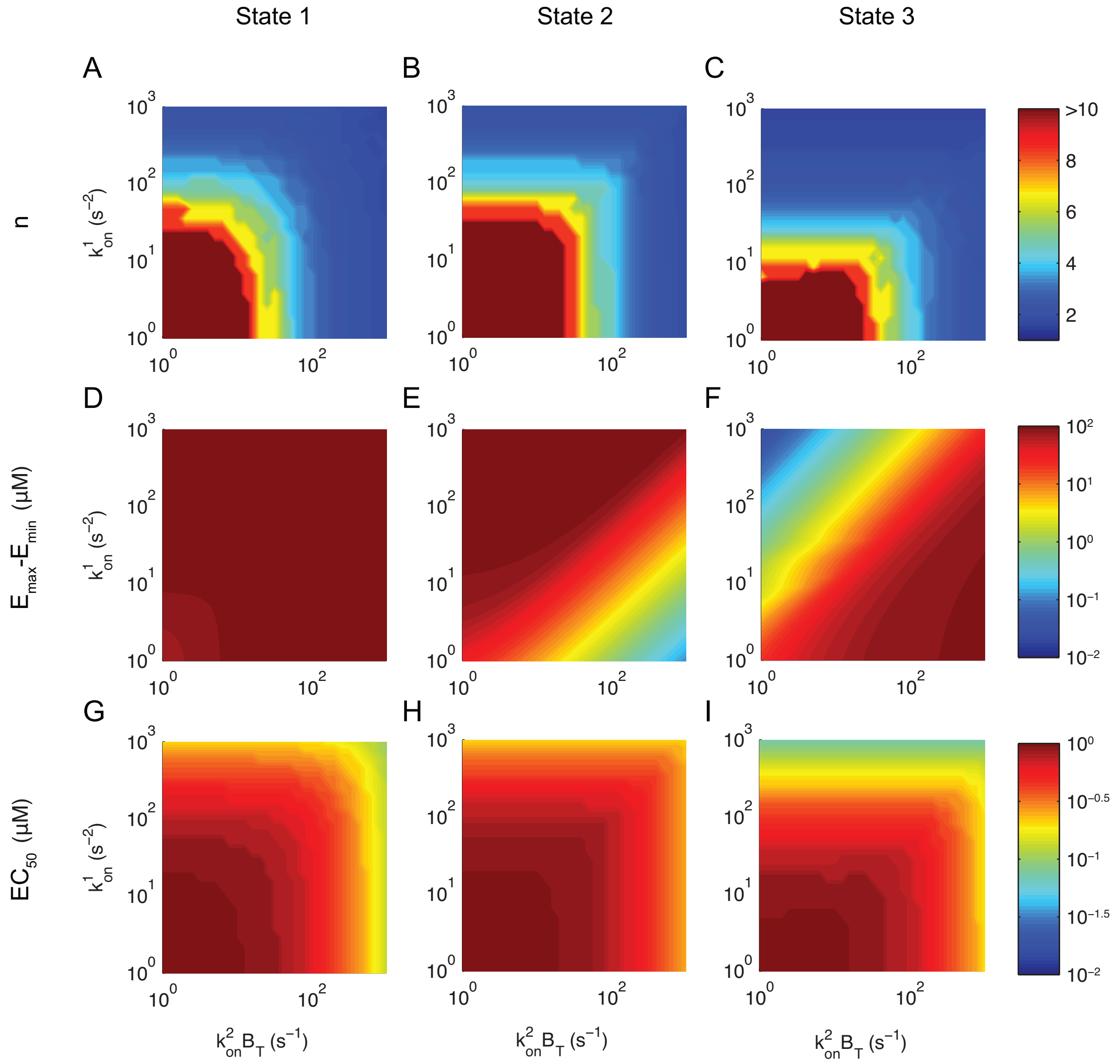

Fig S4: Biosensors exhibiting both intramolecular and intermolecular reactions do not exhibit cooperativity and introduce complex FRET states. When both intramolecular and intermolecular reactions are appreciable, at least three distinct FRET states are possible: a state where the binding domain and the binding motif are both free (State 1), 2) a state when the binding domain is intramolecularly bound to the binding motif (State 2), or 3) a state where the binding domain and/or binding motif is bound to another biosensor, possibly generating large oligomers (State 3). Heat maps showing the Hill number (n) for A) State 1, B) State 2, and C) State 3 as a function of the intramolecular (y-axis) and intermolecular (x-axis) binding rate constants. Note that when, for example, k^1_{on} is large and k^2_{on} is small the concentration of biosensor in oligomers is small. By examining State 1, which is always present, we find that there is no overt cooperativity between intramolecular and intermolecular reactions in reducing the Hill number. Heat maps for D-F) dynamic range and G-I) potency are also shown. Default parameters: [Biosensor]= $B_{\text{T}} = 100\ \mu$M, $F_{\text{T}} = 1\ \mu$M, $k^1_{\text{off}} = 1$ s^{-1}, $k^2_{\text{off}} = 1\ \text{s}^{-1}$, $k^e_{\text{on}} = k^f_{\text{on}} = 10\ \mu\text{M}^{-1}\text{s}^{-1}$, $k^e_{\text{off}} = k^e_{\text{off}} = 1\ \text{s}^{-1}$, $k^e_{\text{cat}} = k^f_{\text{cat}} = 1\ \text{s}^{-1}$.

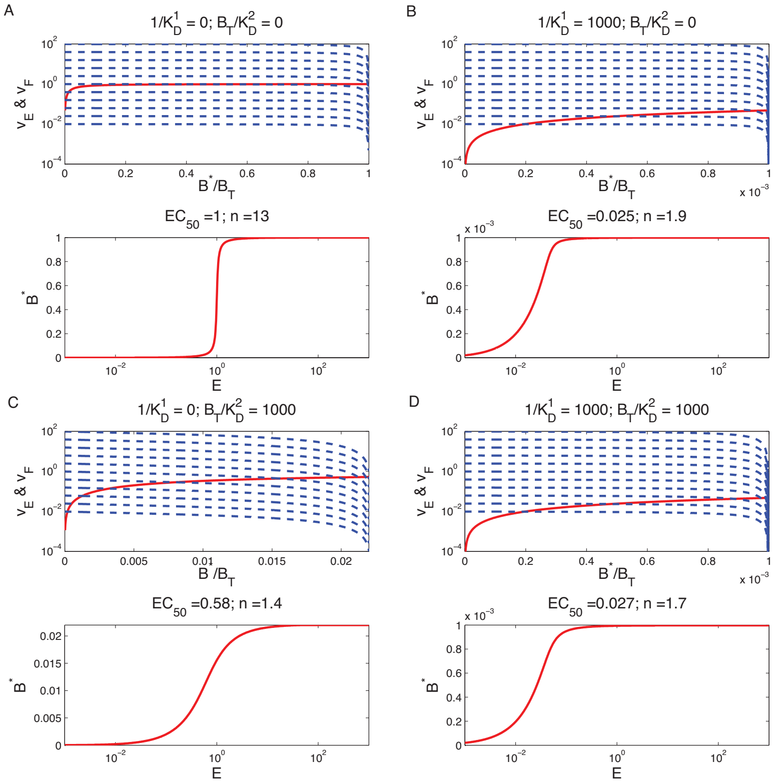

Fig S5: Intramolecular and intermolecular interactions sequester free biosensor, reducing enzyme saturation and ultrasensitivity. Kinase (dashed blue lines) and phosphatase (red solid line) activity as a function of B^*/B_T based on the approximative analytical solution (see text in Supporting Materials) for A) no intramolecular and intermolecular interaction, B) only intramolecular interaction, C) only intermolecular interaction, and D) both intramolecular and intermolecular interactions. The intersection of the kinase and phosphatase activity curves for different kinase levels determines the equilibrium value of B^*, which is plotted as a function of the kinase concentration in the lower part of each panel. The Hill number (n) and potency (EC_{50}) are also indicated. The intramolecular interaction reduces the maximum B^* (compared x-axis scales) by sequestering biosensors in B_c^*, which reduces the ability of the phosphatase to operate in the zero-order regime and hence shifts EC_{50}. Intermolecular interactions are less effective in reducing maximum B^* (compare x-axis scales), but nevertheless is able to reduce the ability of both enzymes to operate in the zero-order regime which reduces ultrasensitivity while only introducing small changes in EC_{50}. No cooperativity between intramolecular and intermolecular interactions is observed in reducing Hill numbers. Parameters: [Biosensor]= $B_\mathrm{T} = 10\ \mu$M, $F_\mathrm{T} = 1\ \mu$M, $k_\mathrm{on}^1 = 0$ s^{-1}, $k_\mathrm{off}^1 = 1$ s^{-1}, $k_\mathrm{on}^2 = 10\ \mu$M^{-1}s^{-1}, $k_\mathrm{off}^2 = 1$ s^{-1}, $k_\mathrm{on}^e = k_\mathrm{on}^f$ =10 μM^{-1}s^{-1}, $k_\mathrm{off}^e = k_\mathrm{off}^e = 1$ s^{-1}, $k_\mathrm{cat}^e = k_\mathrm{cat}^f = 1$ s^{-1}.

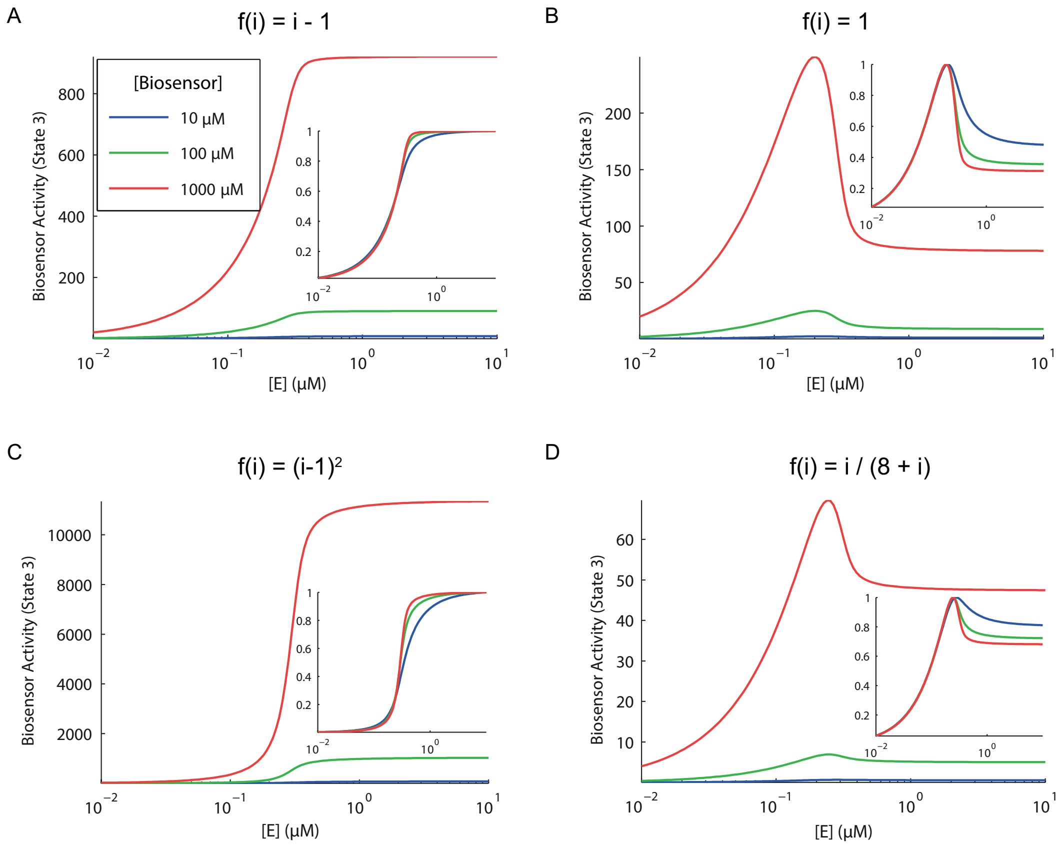

Fig S 6: Biosensor activity strongly depends on the FRET kernel. The biosensor activity arising from a biosensor oligomer will depend on the oligomer size. Therefore we compute the biosensor activity as $\sum_{i=2}^{N} f(i)(B_i^* + B_i)$ where $f(i)$ is the FRET kernel. B_i^* and B_i are the concentration of biosensor in an oligomer of size i whose free tyrosine is phosphorylated or unphosphorylated, respectively. A-D) The biosensor activity is shown for the indicated FRET kernel but with otherwise identical parameters. It is evident that the FRET kernel can have a dramatic impact on the relationship between the enzyme and biosensor activity. Parameters: $F_{\mathrm{T}} = 1\ \mu$M, $k^1_{\mathrm{on}} = 0\ \mathrm{s}^{-1}$, $k^1_{\mathrm{off}} = 1\ \mathrm{s}^{-1}$, $k^2_{\mathrm{on}} = 10\ \mu\mathrm{M}^{-1}\mathrm{s}^{-1}$, $k^2_{\mathrm{off}} = 1\ \mathrm{s}^{-1}$, $k^e_{\mathrm{on}} = k^f_{\mathrm{on}} = 10\ \mu\mathrm{M}^{-1}\mathrm{s}^{-1}$, $k^e_{\mathrm{off}} = k^e_{\mathrm{off}} = 1\ \mathrm{s}^{-1}$, $k^e_{\mathrm{cat}} = k^f_{\mathrm{cat}} = 1\ \mathrm{s}^{-1}$.

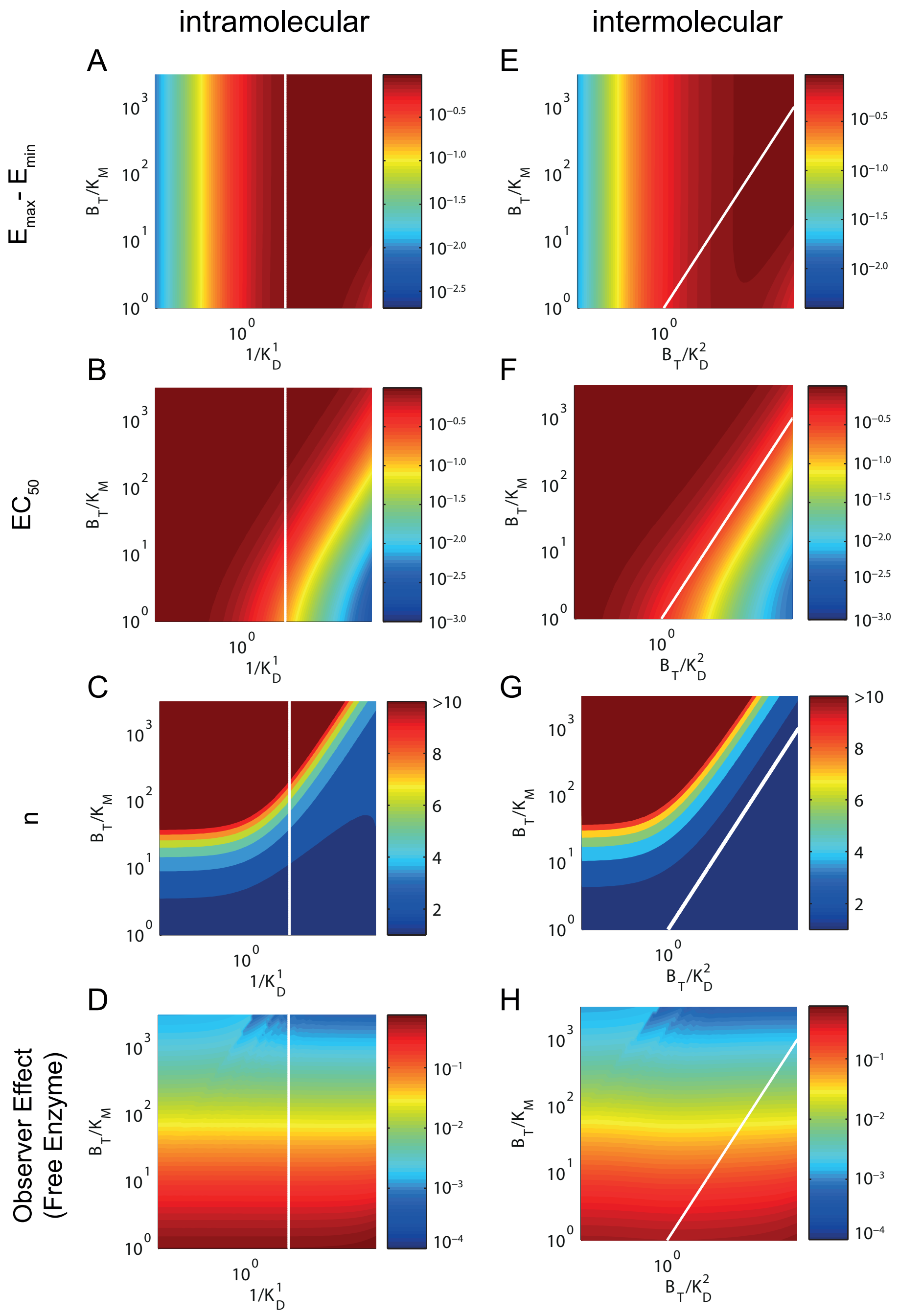

Fig S7: Non-dimensional phase diagrams of biosensor response curve measures. Results are shown for an A-D) exclusively intramolecular biosensor and E-H) exclusively intermolecular biosensor where the maximum biosensor oligomer is of size 2 (dimer). The dynamic range (A,E), potency (B,F), sensitivity (C,G), and observer effect (D,H) are as defined in the main text. White lines illustrate changes in the biosensor concentration (A-D: $K_M = 0.1$ μM and $K_D^1 = 0.1$, E-H) $K_M = K_D^2 = 0.1$ μM). Note that all heat map values are derived directly from non-dimensional calculations (e.g. EC_{50} is the ratio of E_T/F_T at half maximal dynamic range, $(E_{max} - E_{min})/2$, where E_{max} and E_{min} are the maximum and minimum fractions of active biosensor).
