# Supplementary material for: Biosensor Architectures for High-Fidelity Reporting of Cellular Signaling
Source: Biophys J. 2014 Aug 5;107(3):773–82. doi: 10.1016/j.bpj.2014.06.021 (PMC4129486; doi:10.1016/j.bpj.2014.06.021)
Supplement: Document S2. Article plus Supporting Material [file mmc2.pdf]

Article

Biosensor Architectures for High-Fidelity Reporting of Cellular Signaling

Omer Dushek,[1,2,*] Annemarie C. Lellouch,[3,4,5] David J. Vaux,[1] and Vahid Shahrezaei[6,*]
[1]Sir William Dunn School of Pathology and [2]Wolfson Centre for Mathematical Biology, Mathematical Institute, University of Oxford, United Kingdom; [3]Aix Marseille Université, Laboratoire d'Adhésion et Inflammation, Marseille, France; [4]Institut National de la Santé et de la Recherche Médicale U1067, Marseille, France; [5]Centre National de la Recherche Scientifique UMR 7333, Marseille, France; and [6]Department of Mathematics, Imperial College London, United Kingdom

ABSTRACT Understanding mechanisms of information processing in cellular signaling networks requires quantitative measurements of protein activities in living cells. Biosensors are molecular probes that have been developed to directly track the activity of specific signaling proteins and their use is revolutionizing our understanding of signal transduction. The use of biosensors relies on the assumption that their activity is linearly proportional to the activity of the signaling protein they have been engineered to track. We use mechanistic mathematical models of common biosensor architectures (single-chain FRET-based biosensors), which include both intramolecular and intermolecular reactions, to study the validity of the linearity assumption. As a result of the classic mechanism of zero-order ultrasensitivity, we find that biosensor activity can be highly nonlinear so that small changes in signaling protein activity can give rise to large changes in biosensor activity and vice versa. This nonlinearity is abolished in architectures that favor the formation of biosensor oligomers, but oligomeric biosensors produce complicated FRET states. Based on this finding, we show that high-fidelity reporting is possible when a single-chain intermolecular biosensor is used that cannot undergo intramolecular reactions and is restricted to forming dimers. We provide phase diagrams that compare various trade-offs, including observer effects, which further highlight the utility of biosensor architectures that favor intermolecular over intramolecular binding. We discuss challenges in calibrating and constructing biosensors and highlight the utility of mathematical models in designing novel probes for cellular signaling.

INTRODUCTION

The major challenge in studying cellular signaling is no longer to identify new signaling proteins but to understand the interaction networks that the identified signaling proteins form. This is particularly important in various pathologies, such as cancer, where mutations to specific signaling proteins perturb the cellular signaling network, resulting in dysregulated cell proliferation. The development of mechanistic models of cellular signaling relies on quantitative data of the activity of specific signaling proteins in living cells. Fluorescent biosensors, either genetically encodable or synthetic peptide/protein molecular probes, are emerging as important tools for the direct study of signaling protein activity in both healthy and disease cells (1–5). In contrast to conventional tools that are destructive or provide indirect measures of protein activity (e.g., protein localization or phosphorylation state), biosensors provide direct, real-time and -space measurements of signaling protein activity.

Several biosensor architectures have been developed to study the activity of signaling proteins such as proteases, small GTPases, and protein kinases (1–5). A widely employed architecture is a single-chain intramolecular Förster resonance energy transfer (FRET)-based probe, which is exemplified by biosensors developed to study the activity of protein tyrosine kinases ((Fig. 1 *A*), and see, for example, the literature (6–9), the referenced database maintained by Okumoto et al. (4), and the list of kinase probes described in Nhu Ngoc Van and Morris (5)). These biosensors contain donor and acceptor fluorescent proteins connected by a flexible peptide element that links an amino-acid sequence containing a tyrosine residue to a recognition domain, such as a Src homology 2 (SH2) domain, which recognizes the tyrosine residue when phosphorylated.

The sequence flanking the tyrosine residue is designed so that it is specifically phosphorylated by the tyrosine kinase of interest (signaling protein). Phosphorylation of this residue allows for intramolecular binding with the SH2 domain, but also possible is intermolecular binding leading to the formation of oligomeric biosensor chains (Fig. 1 *A*). Both intramolecular and intermolecular interactions are expected to change the relative proximity and/or orientation of the acceptor and donor fluorescent proteins, leading to alteration in FRET efficiency. In this way, measurements of the biosensor FRET efficiency are expected to provide direct information on the activity of the signaling protein of interest (10).

Biosensor activity (FRET efficiency) is routinely used as a quantitative proxy for the activity of a signaling protein. It has been used to determine the activity of a signaling protein

Submitted March 6, 2014, and accepted for publication June 10, 2014.

*Correspondence: omer.dushek@path.ox.ac.uk or v.shahrezaei@imperial.ac.uk

This is an open access article under the CC BY license (http://creativecommons.org/licenses/by/3.0/).

Editor: Charles Wolgemuth.

© 2014 The Authors

0006-3495/14/08/0773/10 $2.00

http://dx.doi.org/10.1016/j.bpj.2014.06.021

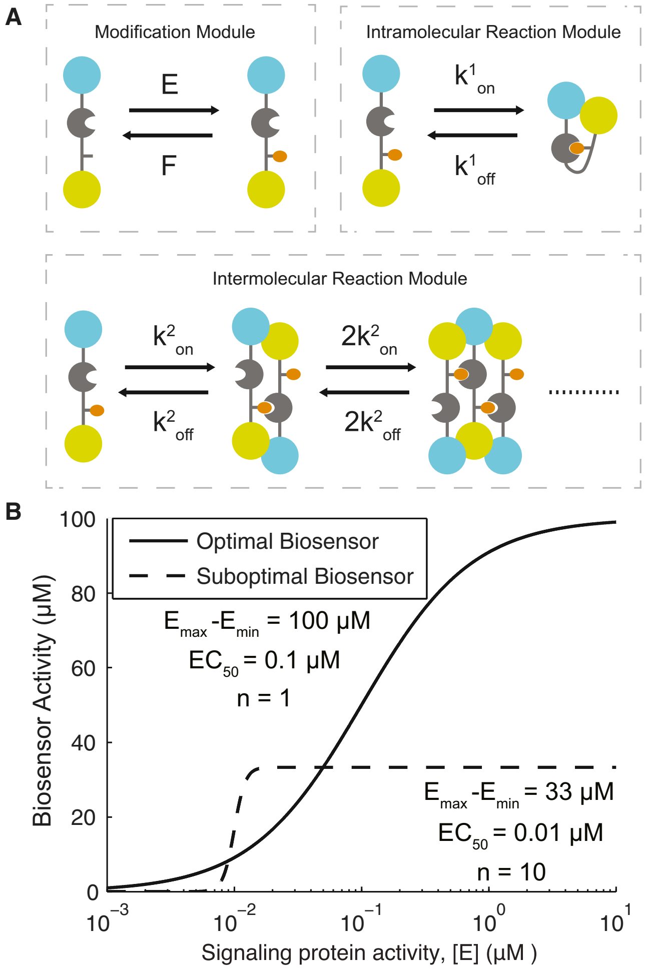

FIGURE 1 Canonical biosensor architectures. (*A*) Architecture of a single-chain FRET-based biosensor with acceptor/donor fluorescent proteins (*cyan*, *yellow*), a recognition domain (*gray circle*), and a modification site that is able to bind the recognition domain when modified (*orange*) by the signaling protein of interest (*E*). For clarity, we use the specific example of a biosensor designed to track the activity of a tyrosine kinase and therefore the modification site is a tyrosine residue and the binding domain an SH2 domain. When phosphorylated, the biosensor is capable of undergoing intramolecular and intermolecular binding reactions, forming a closed monomeric state and oligomeric chain states, respectively. Note that only a small subset of all possible intermolecular reactions are shown. (*B*) Hypothetical biosensor response curves showing the relationship between the concentration of active signaling protein (*E*) and the biosensor activity for an optimal and suboptimal biosensor. The suboptimal biosensor will not reliably report the activity of the signaling protein because it exhibits a threshold (or switchlike response) that saturates at low concentrations of the signaling protein. To see this figure in color, go online.

in different regions of a cell (6–8,11,12), in response to different stimulating conditions (13), and it is expected to be of great benefit for clinical diagnostics and high throughput drug screening (9,14).

The use of biosensors in quantitative studies relies on the assumption that the biosensor activity is approximately linearly proportional to the activity of the signaling protein. It is reasonable to assume that the activity of the biosensor and signaling protein will be related by a saturating sigmoidal curve and in Fig. 1 *B*, we show two hypothetical biosensor response curves. It is clear that an optimal biosensor is one that responds gradually to changes in the signaling protein over relevant signaling protein concentrations, ultimately reaching a large maximum, whereas a suboptimal biosensor is one whose activity does not reflect changes in the activity of the signaling protein.

We note that a switchlike biosensor response curve may be desirable if the only information required is whether the signaling protein exceeds a defined threshold. These intuitive observations can be quantified by three key parameters of sigmoidal curves: the dynamic range (E_{max}–E_{min}, which is the total change in biosensor signal); the potency (EC_{50}, which is the concentration of signaling protein producing half-maximal biosensor activity); and the Hill number (n, which quantifies the sensitivity of the biosensor to the signaling protein). Ideal biosensors should exhibit Hill numbers close to unity ($n \approx 1.0$) to avoid highly nonlinear (or sublinear) response curves, EC_{50} values comparable to the physiological signaling protein concentration to avoid biosensor activity saturation at low concentrations of active signaling protein, and a large dynamic range to increase the experimental signal/noise. Furthermore, it is important that the biosensor response curve is independent of the biosensor concentration so that results can be compared despite differential expression of the biosensor between different cells or between cellular compartments within a single cell. Biosensors with these properties are expected to provide high-fidelity reporting. To the best of our knowledge, the response curve of biosensors has not been quantitatively determined in vivo, and therefore the degree of reporting errors in existing biosensors is unknown.

In this study, we use mechanistic models of single-chain biosensors to study how the biosensor architecture and various reaction parameters determine the biosensor response curve. We find that the canonical biosensor architecture, which works primarily through intramolecular reactions, produces biosensor response curves that depend on the biosensor concentration and that can be highly ultrasensitive (with large Hill numbers), and therefore canonical biosensors may be providing highly nonlinear reporting. The mechanism underlying this nonlinearity is the classic mechanism of zero-order ultrasensitivity (15). In contrast, we find that biosensors operating primarily through intermolecular reactions produce response curves that exhibit small Hill numbers and are concentration-independent. Based on these results, we propose that a single-chain biosensor that permits intermolecular but not intramolecular reactions is likely to provide high-fidelity reporting. The work highlights the importance of calibrating biosensors before use and demonstrates the utility of this mechanistic framework in the design of future novel probes for the study of cellular signaling.

METHODS

Mathematical model

The mathematical model consists of a system of coupled nonlinear ordinary differential equations (ODEs) that are generated in BIONETGEN (16) and integrated in the software MATLAB (The MathWorks, Natick, MA). The complete model consists of 35 distinct chemical species, 330 reactions, and 10 reaction parameters (Table 1). A BIONETGEN file used to generate the ODE system can be found in the Supporting Material.

We model the interactions among the kinase (E), the phosphatase (F), and the biosensor (B) using classical biochemical binding and catalysis reactions. The modification module includes the ability of the kinase to phosphorylate and the phosphatase to dephosphorylate the biosensor whenever it is free and the phosphorylation site accessible (i.e., not bound by an SH2 domain). These enzymatic reactions are as follows:

$$E + B \underset{k^e_{off}}{\overset{k^e_{on}}{\rightleftharpoons}} C \overset{k^e_{cat}}{\rightarrow} E + B^*,$$

$$F + B^* \underset{k^f_{off}}{\overset{k^f_{on}}{\rightleftharpoons}} C^* \overset{k^f_{cat}}{\rightarrow} F + B,$$

where C and C^* represent the kinase-biosensor and phosphatase-biosensor complex, respectively, and B^* represents the phosphorylated biosensor state.

Upon biosensor phosphorylation, the biosensor SH2 domain or SH2 domains on other biosensors can bind the phosphorylated tyrosine. We describe these two reaction types as two modules (Fig. 1). The intramolecular module includes only the interaction between an SH2 domain and a phosphorylated tyrosine on a single biosensor, and is modeled by the following first-order reaction:

$$B^* \underset{k^1_{off}}{\overset{k^1_{on}}{\rightleftharpoons}} B^*_c,$$

where B_c* represents the closed biosensor conformation where the SH2 domain is bound to the phosphorylated tyrosine. The intramolecular binding rates ($k^1{}_{on}$, $k^1{}_{off}$) are both first-order and have units of s^{-1}. The intermolecular module includes bimolecular reactions between biosensors that allow the aggregation of biosensors into oligomeric chains. The number of possible chemical states and reactions are large and therefore the model is described using the following representative reactions:

TABLE 1 Parameter definitions

Parameter	Description	Units
B_T	Biosensor concentration	μM
E_T	Kinase concentration	μM
F_T	Phosphatase concentration	μM
$k^e{}_{on}$	Kinase-biosensor on-rate	$\mu M^{-1}\ s^{-1}$
$k^e{}_{off}$	Kinase-biosensor off-rate	s^{-1}
$k^e{}_{cat}$	Kinase-biosensor catalytic-rate	s^{-1}
$k^f{}_{on}$	Phosphatase-biosensor on-rate	$\mu M^{-1}\ s^{-1}$
$k^f{}_{off}$	Phosphatase-biosensor off-rate	s^{-1}
$k^f{}_{cat}$	Phosphatase-biosensor catalytic-rate	s^{-1}
$K^1{}_{on}$	Biosensor intramolecular on-rate	s^{-1}
$K^1{}_{off}$	Biosensor intramolecular off-rate	s^{-1}
$K_D{}^1$	Intramolecular dissociation constant ($= k^1{}_{off}/k^1{}_{on}$)	
$k^2{}_{on}$	Biosensor intermolecular on-rate	$\mu M^{-1}\ s^{-1}$
$k^1{}_{off}$	Biosensor intermolecular off-rate	S^{-1}
$K_D{}^2$	Intermolecular dissociation constant ($= k^2{}_{off}/k^2{}_{on}$)	μM

$$B^* + B^* \underset{k^2_{off}}{\overset{k^2_{on}}{\rightleftharpoons}} B^*_2,$$

$$B^* + B^*_i \underset{ik^2_{off}}{\overset{2k^2_{on}}{\rightleftharpoons}} B^*_{i+1},$$

$$B + B^*_i \underset{ik^2_{off}}{\overset{k^2_{on}}{\rightleftharpoons}} B_{i+1},$$

where B_2* is a biosensor of oligomer size 2, $k^2{}_{on}$ is the bimolecular on-rate in units of $\mu M^{-1}\ s^{-1}$, and $k^2{}_{off}$ is a first-order off-rate in units of s^{-1}. B_i* and B_i are biosensors of oligomer size i with the free tyrosine phosphorylated or dephosphorylated, respectively. Biosensor chains have the potential to grow at both ends. At the end with an SH2 domain, only phosphorylated biosensors (B*, B_i*, either free or already in an oligomer) can bind while at the end with a phosphorylated tyrosine, both phosphorylated (B*, B_i*, either free or in an oligomer) and unphosphorylated (B, only free) biosensors can bind. Note that the binding of an unphosphorylated biosensor caps the biosensor oligomer, allowing it to grow only at the SH2 domain end (i.e., binding of B to B_i* forms B_{i+1}). The use of BIONETGEN to model these reactions is crucial in allowing all possible reaction routes so that, for example, biosensor oligomers can dissociate (and associate) in every possible combination without making any model simplifications.

To keep the mathematical model computationally tractable, we make simplifying assumptions to the intermolecular reaction module:

1. We limit the number of biosensors in a single oligomer to 15, but find that the fraction of biosensors in oligomers of size 15 is very small (<1%) for the majority of the parameter space, indicating that the introduced error is small (see Fig. S2 in the Supporting Material).
2. We do not include any chain closure reactions, which are intraoligomer reactions that convert biosensor oligomeric chains into a biosensor oligomeric rings. We omit these reactions because there is no information on how these chain closure reactions depend on the biosensor oligomer size. Calculations with a chain closure reaction that is independent of the oligomer size do not alter the qualitative conclusions (data not shown).
3. We prevent enzymes from catalyzing reactions on oligomeric biosensors, but including such reactions does not change the qualitative results (data not shown).
4. We have assumed that the binding ($k^2{}_{on}$) and unbinding ($k^2{}_{off}$) rate constants depend only on the stoichiometry of the oligomer, but are otherwise independent of the oligomer size (e.g., we do not include cooperativity, so that the on-rate constant increases with the size of the oligomer).

Biosensor activity

We define three major biosensor states on the basis that these three states will exhibit different activities or FRET properties, as follows:

State 1 we define as the free biosensor state, where the SH2 domain and the tyrosine are unbound ($B + B^* + C + C^*$).

State 2 we define as the intramolecular biosensor state, where the SH2 domain is bound to the phosphorylated tyrosine on the same biosensor (B_c*).

State 3 we define as all remaining states, namely the intermolecular biosensor state, where the biosensor is in an oligomer.

Determining the biosensor activity in State 3 is made complicated by the possibility that the FRET properties of the biosensor depend on the

oligomeric state of the biosensor. We define the biosensor activity in State 3 as

$$\sum_{i=2}^{N} f(i)\left(B_i^* + B_i\right),$$

where $f(i)$ is the FRET kernel. In the main text, we use the parsimonious assumption that $f(i) = i - 1$ but explore three alternate FRET kernels in Fig. S4.

RESULTS

Generalized mechanistic model for canonical biosensors

To study canonical biosensors, we constructed a generalized mechanistic mathematical model that includes the essential features common to single-chain biosensors (Fig. 1 *A*). These features include a biosensor (B) that, upon phosphorylation (B^*), is able to undergo an intramolecular conformational change (B_c^*) and/or form biosensor oligomers of size i by intermolecular binding (B_i^*). The on-rates and off-rates are $k^1{}_{on}$ and $k^1{}_{off}$ (intramolecular) and $k^2{}_{on}$ and $k^2{}_{off}$ (intermolecular). The biosensor phosphorylation site is modified by a specific signaling protein of interest, which in this example is a kinase (E), and it is assumed that it is dephosphorylated by a constitutively active phosphatase. The coupled system of ODEs describing these reactions were generated in BIONETGEN (16), a rule-based modeling tool, and consists of 35 chemical species, 330 reactions, and 10 rate constants (see Methods). The model parameters are summarized in Table 1.

Canonical intramolecular biosensors may exhibit highly nonlinear reporting

Canonical biosensors are often designed with the intention that only intramolecular reactions are possible. This is the case when the intramolecular on-rate is much larger than the effective intermolecular binding rate (i.e., $k^1{}_{on} >> k^2{}_{on}B_T$, where B_T is the biosensor concentration). To study biosensors in this limit, we set $k^2{}_{on} = 0\ \mu M^{-1}\ s^{-1}$.

Using this reduced model, we compute the biosensor activity as a function of the active kinase concentration at steady state for small (Fig. 2 *A*) and large (Fig. 2 *B*) intramolecular on-rates. In this model, the biosensor activity is defined as the concentration of biosensor in the intramolecular closed conformation (B_c^*). In the case of a small intramolecular on-rate, the model predicts a highly nonlinear

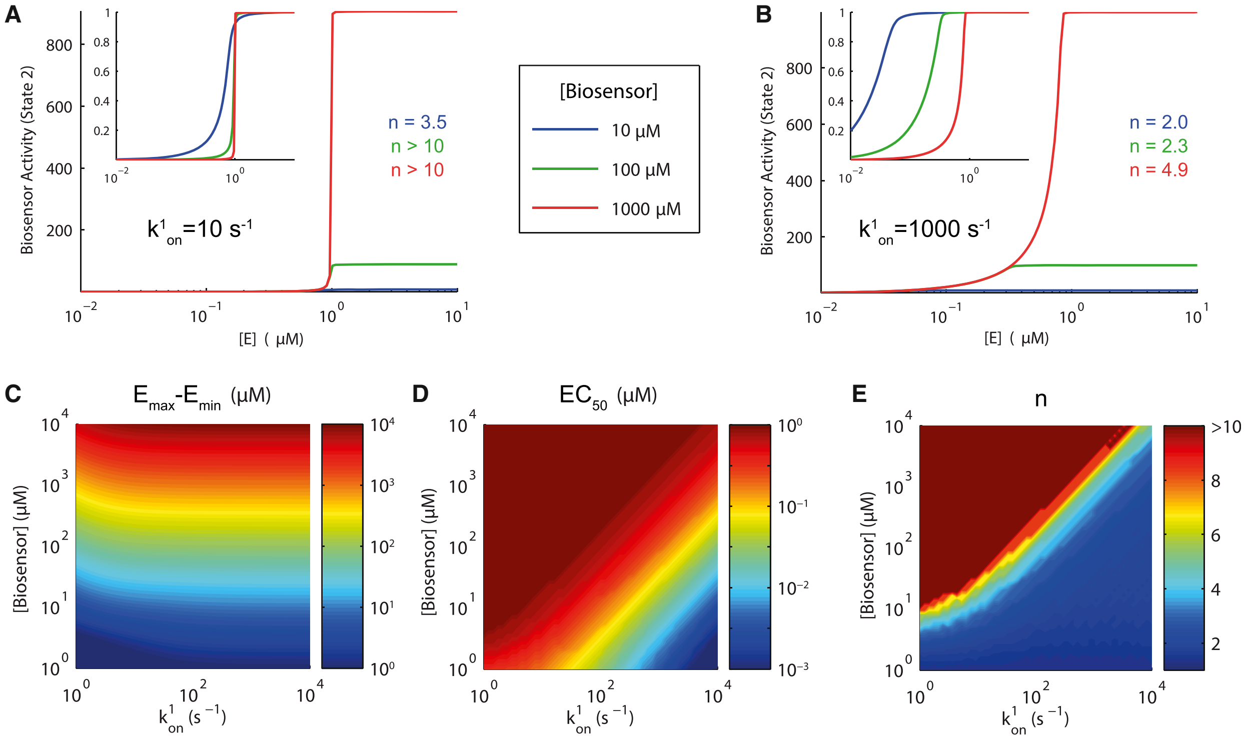

FIGURE 2 Canonical intramolecular biosensors may exhibit highly nonlinear (biosensor concentration-dependent) reporting. (*A* and *B*) Biosensor response curves for three biosensors concentrations when the intramolecular on-rate is (*A*) small and (*B*) large. Highly nonlinear response curves are predicted at large biosensor concentrations, which are readily observed in the normalized curves (*inset*). (*C*–*E*) Heat maps of the dynamic range (E_{max}–E_{min}), potency (EC_{50}), and Hill number (n) of biosensor response curves for different values of the biosensor concentration and intramolecular on-rate highlight that in this architecture the response curves depend on the biosensor concentration and, in general, are highly nonlinear. Parameters: $F_T = 1\ \mu M$, $k^1{}_{off} = 1\ s^{-1}$, $k^2{}_{on} = 0\ \mu M^{-1}\ s^{-1}$, $k^2{}_{off} = 1\ s^{-1}$, $k^e{}_{on} = k^f{}_{on} = 10\ \mu M^{-1}\ s^{-1}$, $k^e{}_{off} = k^e{}_{off} = 1\ s^{-1}$, and $k^e{}_{cat} = k^f{}_{cat} = 1\ s^{-1}$. To see this figure in color, go online.

switchlike (ultrasensitive) relationship between the kinase and biosensor activity, as characterized by large Hill numbers ($n >> 1$). This means that the biosensor activity will remain constant despite large changes in the kinase activity (e.g., when $E < 1$ μM and $E > 1$ μM) and moreover, the biosensor activity may also change dramatically despite only small changes in kinase activity (e.g., $E \approx 1$ μM). In the case of a large intramolecular on-rate, the Hill numbers are reduced but the potency now exhibits a dependence on the biosensor concentration.

These observations are illustrated in heat maps of the dynamic range, potency, and Hill number as a function of $k^1{}_{on}$ and B_T (Fig. 2, *C–E*). We find that the potency remains constant and the dynamic range remains large in the majority of parameter space, which is desirable. However, we find that large Hill numbers are also prevalent in these regions, indicating that canonical biosensors may be providing highly nonlinear reporting. Note that desirable Hill numbers ($n \approx 1$) are possible at low biosensor concentrations or when the intramolecular on-rate is large. However, in the former, the biosensor signal will be undesirably low and in the latter, we observe large changes in potency. Taken together, high-fidelity reporting that is independent of biosensor concentration is not possible in this architecture.

The origin of the observed nonlinearity is zero-order ultrasensitivity, and it operates when opposing enzymes act on a substrate that is in excess of the modifying enzymes (15). Given that the biosensor (substrate) concentration cannot be controlled in vivo, it is likely that it can exceed the concentration of the signaling proteins (modifying enzymes), whose concentration range from 10 nM to 10 μM (17). Because only a fraction of these proteins are catalytically active, biosensor concentrations in excess of these values can result in zero-order ultrasensitivity. It follows that it is experimentally infeasible to avoid zero-order ultrasensitivity. We note that even if the biosensor concentration could be controlled in vivo, it would need to be lower than the enzyme concentrations (~10 nM), making detection difficult. Consistent with the mechanism of zero-order ultrasensitivity, we observe reduction in Hill numbers when the Michaelis-constant (K_M) is large (see Fig. S1).

Attempts to improve biosensors that produce poor reporting (e.g., low signal, no change in signal upon increase in kinase activity, etc.) often involve various modifications to the intramolecular reaction rates (4,18). The intramolecular off-rate ($k^1{}_{off}$) can be modified by altering amino acids in the SH2 domain or those that flank the phosphorylated tyrosine. The intramolecular on-rate ($k^1{}_{on}$) can be manipulated by altering the length of the linker between the SH2 domain and the tyrosine residue. Using our model, we find that increasing the intramolecular affinity reduces Hill numbers (the mechanism of which is discussed below), but comes at a cost to large changes in potency (see Fig. S2). It follows that increasing the fidelity of biosensor reporting cannot be achieved by simply altering the intramolecular reaction rates.

Biosensor oligomerization by intermolecular reactions reduces nonlinear reporting

Before examining the generalized model that allows for both intramolecular and intermolecular reactions, we sought to understand the biosensor response when only intermolecular reactions are possible. Such biosensors can be generated by, for example, placing the phosphorylation site in close proximity to the SH2 domain so that intramolecular reactions are physically not possible but intermolecular reactions are unaffected (e.g., placing the phosphorylation site on the SH2 domain distal from the phosphotyrosine binding pocket). Note that intermolecular reactions allow for the formation of biosensor oligomeric chains, but it is unclear how the FRET properties of the oligomer will depend on chain size. We discuss this issue below, but for now make the parsimonious assumption that the biosensor activity (FRET signal) is linearly proportional to the number of biosensors in the oligomer.

To examine the effects of intermolecular binding alone, we set $k^1{}_{on} = 0$ s^{-1}. Using this reduced model, we compute the biosensor response curve at steady state for small (Fig. 2 *A*) and large (Fig. 2 *B*) intermolecular on-rates. In both cases we find the biosensor response curves are independent of the biosensor concentration and, in the case of a large intermolecular on-rate, we find small Hill numbers. This is further illustrated in heat maps of the dynamic range, potency, and Hill numbers as a function of $k^2{}_{on}$ and B_T (Fig. 3, *C–E*). It is clear that the potency and Hill numbers are independent of the biosensor concentration and the Hill numbers are small when $k^2{}_{on} = 10$ μM s^{-1}. As in the case of a purely intramolecular biosensor, large Hill numbers are a direct result of zero-order ultrasensitivity and can be avoided with a sufficiently large K_M (see Fig. S3). Therefore, high-fidelity reporting is possible by biosensor architectures that exhibit intermolecular but not intramolecular reactions.

Intermolecular and intramolecular reactions reduce ultrasensitivity by sequestering free biosensor

We next examine the biosensor response curves in the most general case when both intramolecular and intermolecular reactions are possible. We note that in this case there are at least three distinct FRET states: all states where the biosensor tyrosine residue and SH2 domain are free (State 1), the single intramolecular binding state (State 2), and all oligomeric states (State 3). To examine the interplay between both reactions, we examined the Hill number of the biosensor response curve as a function of $k^1{}_{on}$ and $k^2{}_{on}B_T$ with a fixed biosensor concentration of 100 μM, and find little synergy in reducing the Hill number between the two reaction modes (see Fig. S4).

To gain insight into the mechanism of how intermolecular and intramolecular reactions reduce ultrasensitivity, we

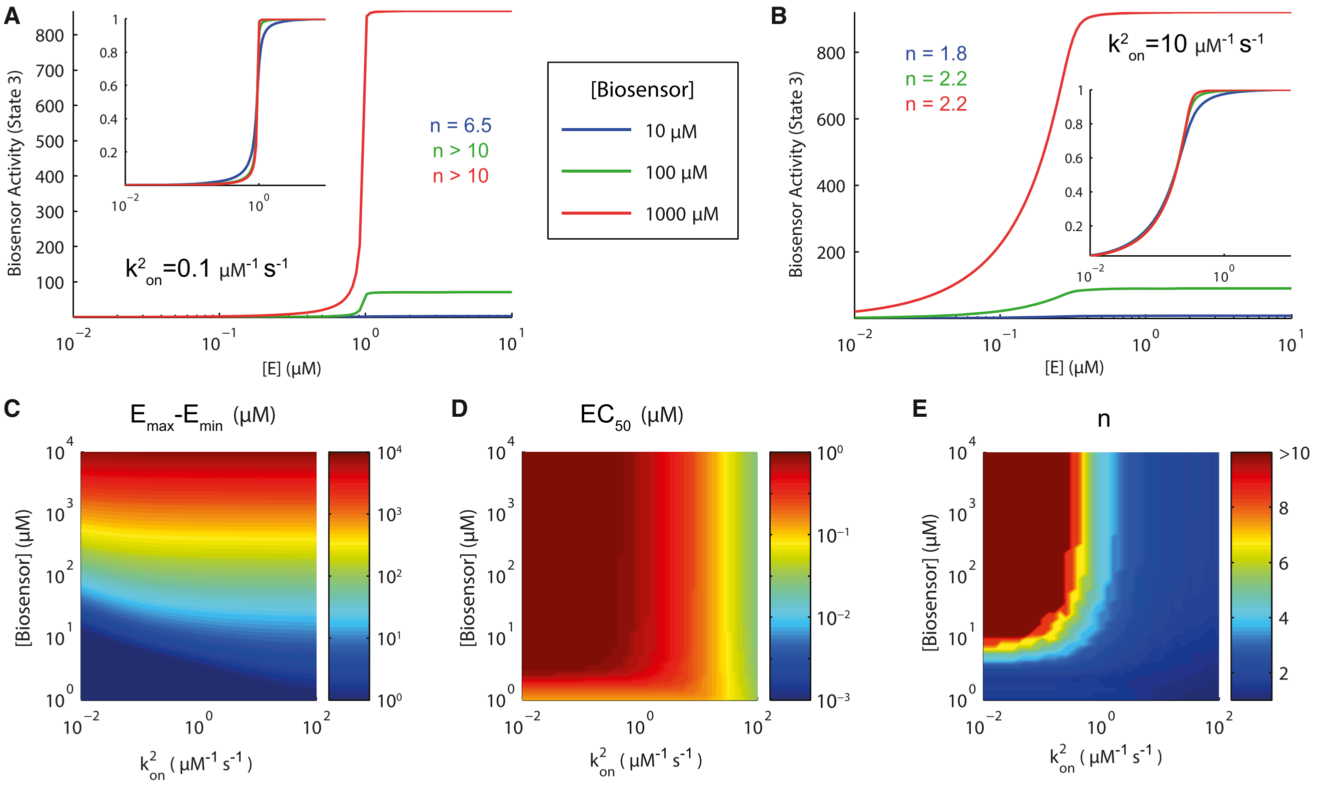

FIGURE 3 Canonical intermolecular biosensors provide improved reporting. (*A* and *B*) Biosensor response curves for three biosensor concentrations when the intermolecular on-rate is (*A*) small or (*B*) large. Observe that a large intermolecular on-rate produces concentration-independent response curves that exhibit reduced Hill numbers. (*C–E*) Heat maps of the dynamic range (E_{max}–E_{min}), potency (EC_{50}), and Hill number (n) of biosensor response curves for different values of the biosensor concentration and intermolecular on-rate highlight that when the intermolecular reaction is $>1\ \mu M^{-1}\ s^{-1}$, the biosensor response curve is independent of the biosensor concentration and exhibits Hill numbers <2. Biosensor activity is assumed to be linearly proportional to the number of biosensors in an oligomer (see Methods for details). Parameters: $F_T = 1\ \mu M$, $k^1{}_{off} = 1\ s^{-1}$, $k^2{}_{on} = 0\ \mu M^{-1}\ s^{-1}$, $k^2{}_{off} = 1\ s^{-1}$, $k^e{}_{on} = k^f{}_{on} = 10\ \mu M^{-1}\ s^{-1}$, $k^e{}_{off} = k^e{}_{off} = 1\ s^{-1}$, and $k^e{}_{cat} = k^f{}_{cat} = 1\ s^{-1}$. To see this figure in color, go online.

have used the graphical approach pioneered by Ferrell (19) (see the Supporting Material). We observe that both intermolecular and intramolecular reactions reduce the ability of the two enzymes to enter the zero-order regime, resulting in lower Hill numbers (see Fig. S5). The reduced ability to enter the zero-order regime is a result of sequestration of free biosensors into states that are shielded from further enzymatic action effectively preventing enzyme saturation, a requirement for zero-order ultrasensitivity (15). For example, the intramolecular reaction that forms B_c* prevents the phosphatase from dephosphorylating the biosensor. This assumption is reasonable because intramolecular and intermolecular reactions are expected to sterically shield the modification sites from catalytic domains (e.g., SH2 domain binding to phosphorylated tyrosines shields them from dephosphorylation (20)).

Canonical biosensors may exhibit complex FRET states

Canonical biosensors that undergo both intramolecular and intermolecular reactions may introduce many distinct FRET states, making it difficult to relate experimental measurements to the concentration of biosensor in specific states and ultimately to the activity of the signaling protein.

As already mentioned, it is presently unknown how the FRET efficiency will depend on the number of biosensors in an oligomeric chain and in addition, the formation of such oligomeric chains may actually alter the absorption and emission spectra of both the donor and acceptor (21,22). Without this information, we have so far assumed that the FRET efficiency will increase linearly with the number of biosensors in an oligomer and this assumption has important consequences for the shape of the biosensor response curve. We highlight this by showing biosensor response curves when alternate (e.g., nonlinear) relationships are assumed, finding that the response curves can exhibit increased Hill numbers or nonmonotonic response curves (see Fig. S6). We note that even if a complete description of the oligomeric FRET states was available, the existences of at least two other distinct FRET states (free and intramolecularly bound) means that relating FRET measurements to specific biosensor states, and ultimately

to the activity of the signaling protein, is not possible without additional information.

Improved biosensor architecture for high-fidelity reporting

Taken together, the results so far suggest that high-fidelity reporting can be accomplished by a biosensor architecture that allows for intermolecular binding. Moreover, to relate the FRET signal to a specific biosensor state, we propose that the biosensor should be restricted to form only dimers and prevented from forming intramolecular reactions. This architecture may be realized by placing the tyrosine residue near the SH2 domain-binding pocket so that intramolecular reactions are physically not possible. Moreover, the formation of biosensor dimers may then sterically occlude other biosensors from interacting with the free SH2 domain or free tyrosine in a dimer, preventing the formation of larger biosensor oligomers.

We next directly compared this biosensor architecture (exclusively intermolecular where the maximum oligomer is a dimer) to the canonical intramolecular architecture (exclusively intramolecular). A mathematical analysis of these simpler architectures (see the Supporting Material) allowed us to identify the ratio of biosensor concentration to the Michaelis-Menten constant (B_T/K_M) and the relative strength of the SH2 domain binding ($1/K_D^1$ for the intramolecular biosensor and B_T/K_D^2 for the intermolecular biosensor) as the two critical nondimensional parameters determining the fidelity of reporting (see the Supporting Material). Phase diagrams comparing the high-fidelity reporting regimes for the two biosensor architectures reveal that high-fidelity reporting, which is independent of biosensor concentration, can only be achieved for the intermolecular architecture (Fig. 4). In this calculation, the ratio of phosphatase to biosensor concentration is fixed at 0.01 and this ratio must be <1 to observe zero-order ultrasensitivity. Although large values of K_M allow the intramolecular biosensor to enter the high-fidelity regime, changes in the biosensor concentration can lead to departures from the regime (*black line*). This is not the case for the intermolecular architecture, which sustains high-fidelity reporting when $K_M \approx K_D^2$, independent of biosensor concentration (*black line*).

Observer effects

Introducing biosensors, or other exogenous protein, into cells is expected to perturb endogenous signaling. Given that the signaling enzyme is explicitly included in our mathematical model, we are able to directly calculate the fraction of the signaling protein that is bound (in an enzyme-substrate complex), and therefore sequestered by the biosensor (not able to act on endogenous substrates). We find that $> 75\%$ of the signaling protein is sequestered by the biosensor in nearly all of parameter space for both architectures (Fig. 4). As expected, the observer effect is lower at higher values of K_M (see also Fig. S7, *D* and *H*).

DISCUSSION

Biosensors are emerging as important tools for the quantitative study of cellular signaling. Their utility is predicated on

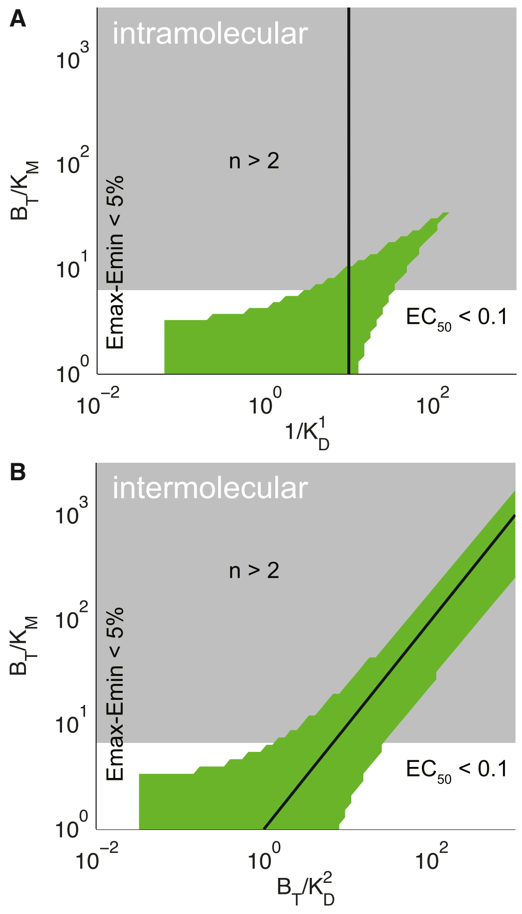

FIGURE 4 High-fidelity regimes (*green*) for the intramolecular and intermolecular biosensor architectures. Architectures represented are (*A*) exclusively intramolecular and (*B*) exclusively intermolecular, with a maximum oligomer of size 2 (dimer). In both cases, the *y* axis shows the ratio of biosensor concentration to the Michaelis-Menten constant and the *x* axis shows the strength of the intramolecular or intermolecular reaction. To the left of the high-fidelity region the biosensor will provide only a weak signal (dynamic range <5%), to the right of the high-fidelity region the biosensor will saturate at low concentration of the signaling protein ($EC_{50} < 0.1$), and above the high-fidelity region the biosensor response curve will be highly nonlinear ($n > 2$). Observer effect, defined by the biosensor sequestering >75% of the signaling enzyme at the EC_{50}, is appreciable over most of parameter space (*gray*). Note that high-fidelity reporting is possible for the intermolecular architecture despite changes in biosensor concentration (indicated by *black lines*), because the biosensor concentration appears in both nondimensional parameters for the intermolecular but not the intramolecular architecture. This result is consistent with heat maps shown in Figs. 2 and 3. See Fig. S7 in the Supporting Material for heat maps of individual biosensor response curve parameters. Generation of these nondimensional panels is described in the Supporting Material. To see this figure in color, go online.

high-fidelity reporting, which requires that biosensor response curves exhibit large dynamic ranges, relevant potencies, and gradual (non-switch-like) sensitivities. Moreover, it is important that high-fidelity reporting is maintained despite fluctuations in the biosensor concentration. We have shown that high-fidelity reporting that is independent of the biosensor concentration can be achieved by an intermolecular but not by an intramolecular architecture.

The general model we considered revealed that biosensors may exhibit response curves that are highly nonlinear, as a result of zero-order ultrasensitivity (15), but that both intramolecular and intermolecular interactions can reduce ultrasensitivity by sequestering the biosensor, which is consistent with previous work (23–25). Only intermolecular interactions produced high-fidelity reporting that is independent of the biosensor concentration (Fig. 3); but these interactions nonetheless highlighted the problem that oligomeric biosensors may have complex FRET states (see Fig. S4). Large oligomeric states may also affect the diffusion coefficient and accessibility of phosphorylation sites, thereby affecting performance of the biosensor. A solution to this is a biosensor architecture that allows for intermolecular, but not intramolecular, interactions and is limited to forming dimers so that only two well-defined FRET states are possible.

In the case of tyrosine kinase biosensors, such an architecture may be achieved by placing the tyrosine residue (with relevant flanking amino acids) on the SH2 domain. Changes in FRET status would result from the intermolecular reaction facilitated by the head-to-tail orientation imposed by the SH2 to substrate interaction. By virtue of the importance of SH2 domains in both receptor tyrosine kinases (26) (e.g., growth factor receptors) and noncatalytic tyrosine-phosphorylated receptors (27) (e.g., immune receptors) signal transduction events, a vast amount of structural data exists for these domains, making them attractive for biosensor engineering (28). The optimal architecture will limit intermolecular interactions to the formation of dimers, which will facilitate interpretation of the FRET signal. Despite recent improvements in fluorescent proteins and high-throughput cloning strategies, biosensor development remains a largely empirical process, and thus specific strategies for achieving dimers will have to be developed for each structure (10). Note that in contrast to other FRET-based intermolecular biosensors, the proposed novel biosensor contains only a single chain maintaining genetic simplicity and fluorescence parity, so that contemporary analytical methods may still be applied.

The mechanism by which many single-chain FRET-based biosensors functions is not completely known. Early tyrosine kinase biosensor work centered on the Abl kinase phosphorylation of the Crk adaptor protein, which was known from extensive structural studies to undergo an intramolecular SH2-phosphotyrosine interaction (6,29,30). However, few biosensors designed since have demonstrated that their constructs function via an intramolecular binding mechanism. Structure-function studies, such as those recently reported for the calcium biosensor TN-XXL (31), in which techniques such as small-angle x-ray scattering and NMR were used to demonstrate the intramolecular nature of the structural changes leading to FRET, would greatly improve future biosensor engineering and validate any alternate architectures, such as the proposed intermolecular biosensor.

Experimental studies aimed at investigating the fidelity of biosensor reporting are presently missing. A theoretical study by Haugh (32) has highlighted several factors that may contribute to low-fidelity reporting by using reaction-diffusion models to explore the limits on measurements of kinetics and spatial gradients during live-cell imaging. We go beyond this study by explicitly modeling the modifying enzymes showing that their dynamics can result in low-fidelity reporting, and we provide an alternate architecture that resolves this issue. This work highlights the need to experimentally calibrate biosensors, which can be performed by measuring the biosensor activity at different concentrations of a chemical inhibitor to the signaling protein. By explicitly including the modifying enzymes, our model is also able to quantify the fraction of signaling protein that is sequestered by the biosensor (Fig. 4). We note that this observer effect is different from the observer effect from a previous report that did not explicitly model the modifying enzymes (32). These observer effects, which likely plague many studies that rely on introducing exogenous proteins into living cells, can be reduced by increasing K_M in both the intramolecular and intermolecular architectures (Fig. 4). Although we have focused on biosensor response curves in steady state, our results are directly applicable to kinetics if the signaling protein changes on timescales that are slower than reaction rates. A detailed analysis of kinetics was provided in Haugh (32).

A recent theoretical study focusing on crosstalk provides relevant insights into biosensor design (33). The authors show that a set of phosphorylated substrates that share kinases and phosphatases will have transitive zero-order ultrasensitivity, meaning that if one of the substrates saturates the enzymes then all substrates will exhibit ultrasensitivity. This implies that a biosensor that is operating in the ultrasensitive regime not only produces poor reporting but may also perturb the dose-response of endogenous substrates. Therefore, our proposed-novel design has the additional benefit of reducing crosstalk with the endogenous system. On the other hand, if the kinase under study is saturated by its endogenous substrates, then the biosensor will produce nonlinear reporting regardless of its design or operating regime. Furthermore, Rowland et al. (33) show that substrate modification can be coupled through shared phosphatases, even if the substrates talk to different kinases. In theory, a solution to these complications is to design the

biosensor so that it does not share a phosphatase with the endogenous substrate but in practice, this would be difficult to implement. A possible workaround would be to include a dominant functional phosphatase domain directly on the biosensor. This would have the added advantage of ensuring that dephosphorylation cannot be saturated and therefore zero-order ultrasensitivity can be avoided.

There is considerable interest in developing biosensors for clinical diagnostics and for drug screens (14). In chronic myeloid leukemia (CML), a mutant fusion gene, *Bcr-abl*, codes for a constitutively active tyrosine kinase, BCR-ABL, whose inhibition by the drug imatinib mesylate (IM) dramatically reduces the cancer burden (34). However, a subset of patients or patients in later stages of CML do not respond to IM, motivating the development of second-generation drugs. Recently, a biosensor aimed at quantifying the activity of BCR-ABL has been developed (9) based on the canonical biosensor architecture (Fig. 2). It is proposed that this biosensor can be used as a diagnostic tool to investigate the activity of BCR-ABL between CML patients and also for high-throughput drug screens for novel BCR-ABL inhibitors.

This work has highlighted the possibility that the BCR-ABL canonical biosensor may exhibit no change in the activity despite large changes in the activity of BCR-ABL, and, vice versa, large changes in the BCR-ABL biosensor may correspond to only modest changes in the activity of BCR-ABL (Fig. 2). It follows that drugs screened based on the activity of the biosensor in this regime may not have the desired functional inhibitory effects. Given the availability of a specific inhibitor for BCR-ABL (e.g., IM), a detailed dose-response should reveal whether the activity of the biosensor is ultrasensitive (highly nonlinear) to the activity of BCR-ABL. Observing an ultrasensitive response is likely and may mean that an improved biosensor architecture, like the one proposed in this work, may be an important step to ensure high-fidelity reporting. These types of validations will also be critical in screening techniques or cellular imaging studies aiming to use multiple biosensors simultaneously or studies aimed at establishing hierarchy in signaling events (2).

Mathematical modeling is commonly used to study information processing in biochemical networks (19,35–38). Predictive modeling and systematic engineering approaches also have contributed significantly to the recent progress in synthetic biology (39–41). Biosensor dynamics and its interaction with the signaling systems are complex and intuition alone may not suffice to assess their behavior and reliability. For example, there can be nontrivial effects due to spatial clustering of large biosensor oligomers (42) or nonmonotonic dose-response in the presence of multiple phosphorylation sites (43). This study highlights the utility of mechanistic mathematical models in the calibration of today's biosensors and also in the design and validation of future novel probes of cellular signaling.

SUPPORTING MATERIAL

Nondimensional analysis, approximate analytical solutions, BioNetGen code used to generate the full model, and seven figures are available at http://www.biophysj.org/biophysj/supplemental/S0006-3495(14)00661-4.

We thank P. Anton van der Merwe and Jesse Goyette for helpful comments.

The work has been supported by the E. P. Abraham Research Fund and by a Sir Henry Dale Fellowship jointly funded by the Wellcome Trust and the Royal Society (grant No. 098363 to O.D.).

REFERENCES

1. Kiyokawa, E., S. Hara, …, M. Matsuda. 2006. Fluorescence (Förster) resonance energy transfer imaging of oncogene activity in living cells. *Cancer Sci.* 97:8–15.
2. Carlson, H. J., and R. E. Campbell. 2009. Genetically encoded FRET-based biosensors for multiparameter fluorescence imaging. *Curr. Opin. Biotechnol.* 20:19–27.
3. Mehta, S., and J. Zhang. 2011. Reporting from the field: genetically encoded fluorescent reporters uncover signaling dynamics in living biological systems. *Annu. Rev. Biochem.* 80:375–401.
4. Okumoto, S., A. Jones, and W. B. Frommer. 2012. Quantitative imaging with fluorescent biosensors. *Annu. Rev. Plant Biol.* 63:663–706.
5. Nhu Ngoc Van, T., and M. C. Morris. 2013. Fluorescent sensors of protein kinases: from basics to biomedical applications. *Prog. Mol. Biol. Transl. Sci.* 113:217–274.
6. Ting, A. Y., K. H. Kain, …, R. Y. Tsien. 2001. Genetically encoded fluorescent reporters of protein tyrosine kinase activities in living cells. *Proc. Natl. Acad. Sci. USA.* 98:15003–15008.
7. Wang, Y., E. L. Botvinick, …, S. Chien. 2005. Visualizing the mechanical activation of Src. *Nature.* 434:1040–1045.
8. Randriamampita, C., P. Mouchacca, …, A. C. Lellouch. 2008. A novel ZAP-70 dependent FRET based biosensor reveals kinase activity at both the immunological synapse and the antisynapse. *PLoS One.* 3:e1521.
9. Mizutani, T., T. Kondo, …, Y. Ohba. 2010. A novel FRET-based biosensor for the measurement of BCR-ABL activity and its response to drugs in living cells. *Clin. Cancer Res.* 16:3964–3975.
10. Fritz, R. D., M. Letzelter, …, O. Pertz. 2013. A versatile toolkit to produce sensitive FRET biosensors to visualize signaling in time and space. *Sci. Signal.* 6:rs12.
11. Yoshizaki, H., Y. Ohba, …, M. Matsuda. 2003. Activity of Rho-family GTPases during cell division as visualized with FRET-based probes. *J. Cell Biol.* 162:223–232.
12. Aoki, K., and M. Matsuda. 2009. Visualization of small GTPase activity with fluorescence resonance energy transfer-based biosensors. *Nat. Protoc.* 4:1623–1631.
13. Aoki, K., N. Komatsu, …, M. Matsuda. 2012. Stable expression of FRET biosensors: a new light in cancer research. *Cancer Sci.* 103:614–619.
14. Lu, S., and Y. Wang. 2010. Fluorescence resonance energy transfer biosensors for cancer detection and evaluation of drug efficacy. *Clin. Cancer Res.* 16:3822–3824.
15. Goldbeter, A., and D. E. Koshland, Jr. 1981. An amplified sensitivity arising from covalent modification in biological systems. *Proc. Natl. Acad. Sci. USA.* 78:6840–6844.
16. Faeder, J. R., M. L. Blinov, and W. S. Hlavacekc. 2009. Rule-based modeling of biochemical systems with BIONETGEN. *In* Methods in Molecular Biology: Systems Biology Humana Press, Totowa, NJ.
17. Milo, R., P. Jorgensen, …, M. Springer. 2010. BioNumbers—the database of key numbers in molecular and cell biology. *Nucleic Acids Res.* 38:D750–D753.

18. Hires, S. A., Y. Zhu, and R. Y. Tsien. 2008. Optical measurement of synaptic glutamate spillover and reuptake by linker optimized glutamate-sensitive fluorescent reporters. *Proc. Natl. Acad. Sci. USA.* 105:4411–4416.

19. Ferrell, Jr., J. E. 1996. Tripping the switch fantastic: how a protein kinase cascade can convert graded inputs into switch-like outputs. *Trends Biochem. Sci.* 21:460–466.

20. Rotin, D., B. Margolis, …, J. Schlessinger. 1992. SH2 domains prevent tyrosine dephosphorylation of the EGF receptor: identification of Tyr^{992} as the high-affinity binding site for SH2 domains of phospholipase C γ. *EMBO J.* 11:559–567.

21. Moss, F. J., P. I. Imoukhuede, …, H. A. Lester. 2009. GABA transporter function, oligomerization state, and anchoring: correlates with subcellularly resolved FRET. *J. Gen. Physiol.* 134:489–521.

22. Albizu, L., M. Cottet, …, T. Durroux. 2010. Time-resolved FRET between GPCR ligands reveals oligomers in native tissues. *Nat. Chem. Biol.* 6:587–594.

23. Ortega, F., L. Acerenza, …, M. Cascante. 2002. Product dependence and bifunctionality compromise the ultrasensitivity of signal transduction cascades. *Proc. Natl. Acad. Sci. USA.* 99:1170–1175.

24. Blüthgen, N., F. J. Bruggeman, …, B. N. Kholodenko. 2006. Effects of sequestration on signal transduction cascades. *FEBS J.* 273:895–906.

25. Salazar, C., and T. Höfer. 2007. Versatile regulation of multisite protein phosphorylation by the order of phosphate processing and protein-protein interactions. *FEBS J.* 274:1046–1061.

26. Lemmon, M. A., and J. Schlessinger. 2010. Cell signaling by receptor tyrosine kinases. *Cell.* 141:1117–1134.

27. Dushek, O., J. Goyette, and P. A. van der Merwe. 2012. Non-catalytic tyrosine-phosphorylated receptors. *Immunol. Rev.* 250:258–276.

28. Kraskouskaya, D., E. Duodu, …, P. T. Gunning. 2013. Progress towards the development of SH2 domain inhibitors. *Chem. Soc. Rev.* 42:3337–3370.

29. Rosen, M. K., T. Yamazaki, …, L. E. Kay. 1995. Direct demonstration of an intramolecular SH2-phosphotyrosine interaction in the Crk protein. *Nature.* 374:477–479.

30. Cotton, G. J., and T. W. Muir. 2000. Generation of a dual-labeled fluorescence biosensor for Crk-II phosphorylation using solid-phase expressed protein ligation. *Chem. Biol.* 7:253–261.

31. Geiger, A., L. Russo, …, O. Griesbeck. 2012. Correlating calcium binding, Förster resonance energy transfer, and conformational change in the biosensor TN-XXL. *Biophys. J.* 102:2401–2410.

32. Haugh, J. M. 2012. Live-cell fluorescence microscopy with molecular biosensors: what are we really measuring? *Biophys. J.* 102:2003–2011.

33. Rowland, M. A., W. Fontana, and E. J. Deeds. 2012. Crosstalk and competition in signaling networks. *Biophys. J.* 103:2389–2398.

34. Druker, B. J. 2008. Translation of the Philadelphia chromosome into therapy for CML. *Blood.* 112:4808–4817.

35. Salazar, C., and T. Höfer. 2009. Multisite protein phosphorylation—from molecular mechanisms to kinetic models. *FEBS J.* 276:3177–3198.

36. Dushek, O., P. A. van der Merwe, and V. Shahrezaei. 2011. Ultrasensitivity in multisite phosphorylation of membrane-anchored proteins. *Biophys. J.* 100:1189–1197.

37. Kholodenko, B., M. B. Yaffe, and W. Kolch. 2012. Computational approaches for analyzing information flow in biological networks. *Sci. Signal.* 5:re1.

38. Prabakaran, S., G. Lippens, …, J. Gunawardena. 2012. Post-translational modification: nature's escape from genetic imprisonment and the basis for dynamic information encoding. *Interdiscip. Rev. Syst. Biol. Med.* 4:565–583.

39. Marchisio, M. A., and J. Stelling. 2009. Computational design tools for synthetic biology. *Curr. Opin. Biotechnol.* 20:479–485.

40. Koide, T., W. L. Pang, and N. S. Baliga. 2009. The role of predictive modeling in rationally re-engineering biological systems. *Nat. Rev. Microbiol.* 7:297–305.

41. Slusarczyk, A. L., A. Lin, and R. Weiss. 2012. Foundations for the design and implementation of synthetic genetic circuits. *Nat. Rev. Genet.* 13:406–420.

42. Mugler, A., A. G. Bailey, …, P. R. ten Wolde. 2012. Membrane clustering and the role of rebinding in biochemical signaling. *Biophys. J.* 102:1069–1078.

43. Suwanmajo, T., and J. Krishnan. 2013. Biphasic responses in multi-site phosphorylation systems. *J. R. Soc. Interface.* 10:20130742.

Supporting Materials:
Biosensor architectures for high fidelity reporting of cellular signaling

Omer Dushek[1,2,¶], Annemarie C Lellouch[3,4,5], David J. Vaux[1], Vahid Shahrezaei[6,¶]

[1]Sir William Dunn School of Pathology, University of Oxford, UK
[2]Wolfson Centre for Mathematical Biology, Mathematical Institute, University of Oxford, UK
[3]Aix Marseille Université, Laboratoire d'Adhésion et Inflammation, Marseille, 13288, France
[4]Inserm U1067, Marseille, 13288, France
[5]CNRS UMR 7333, Marseille, 13288, France
[6]Department of Mathematics, Imperial College London, UK
[¶]Corresponding authors

Non-dimensional analysis of a reduced model

We consider a reduced model that includes both intramolecular and intermolecular reactions but introduce the simplification that the maximum oligomer that can be formed is a dimer. In this reduced model all possible reactions can be enumerated as follows,

$$E + B \underset{k_{\text{off}}^{e}}{\overset{k_{\text{on}}^{e}}{\rightleftharpoons}} C \overset{k_{\text{cat}}^{e}}{\rightharpoonup} E + B^*$$

$$F + B^* \underset{k_{\text{off}}^{f}}{\overset{k_{\text{on}}^{f}}{\rightleftharpoons}} D \overset{k_{\text{cat}}^{f}}{\rightharpoonup} F + B$$

$$B^* \underset{k_{\text{off}}^{1}}{\overset{k_{\text{on}}^{1}}{\rightleftharpoons}} B_C^*, \quad B^* + B^* \underset{k_{\text{off}}^{2}}{\overset{k_{\text{on}}^{2}}{\rightleftharpoons}} B_2^*, \quad B^* + B \underset{k_{\text{off}}^{2}}{\overset{k_{\text{on}}^{2}}{\rightleftharpoons}} B_2$$

The corresponding non-dimensional steady-state equations are:

$$\lambda_1 \widehat{C} = \widehat{B}\widehat{E}$$
$$\lambda_2 \widehat{D} = \widehat{B}^* \widehat{F}$$
$$\lambda_3 \widehat{B}^*_C = \widehat{B}^*$$
$$\lambda_4 \widehat{B}^*_2 = 2(\widehat{B}^*)^2$$
$$\lambda_4 \widehat{B}_2 = 2\widehat{B}^* \widehat{B}$$
$$\lambda_5 \lambda_7 \widehat{C} = \widehat{D}$$
$$1 = \widehat{E} + \widehat{C}$$
$$1 = \widehat{F} + \widehat{D}$$
$$1 = \widehat{B} + \widehat{B}^* + \lambda_6 \lambda_7 \widehat{C} + \lambda_6 \widehat{D} + \widehat{B}^*_C + (\widehat{B}^*_2 + \widehat{B}_2)$$

where 'hat' quantities are the non-dimensional concentrations and the λ_i are the non-dimensional parameters defined as follows: $\widehat{E} = E/E_T$, $\widehat{F} = F/F_T$, $\widehat{C} = C/E_T$, $\widehat{D} = D/F_T$, $\widehat{B} = B/B_T$, $\widehat{B^*} = B^*/B_T$, $\widehat{B^*_C} = B^*_C/B_T$, $\widehat{B_2} = B_2/(B_T/2)$,$\widehat{B^*_2} = B^*_2/(B_T/2)$,$\lambda_1 = K^e_{\text{M}}/B_T$, $\lambda_2 = K^f_{\text{M}}/B_T$, $\lambda_3 = K^1_{\text{D}}$, $\lambda_4 = K^2_{\text{D}}/B_T$, $\lambda_5 = k^e_{\text{cat}}/k^f_{\text{cat}}$, $\lambda_6 = F_T/B_T$, and $\lambda_7 = E_T/F_T$. We make the simplification that the binding and catalytic activities are identical for the two enzymes ($\lambda_1 = \lambda_2$, $\lambda_5 = 1$) which reduces the number of non-dimensional parameters to 5.

We generate biosensor response curves by solving the system of equations using *fsolve* in Matlab (Mathework, MA) for increasing values of λ_7 whilst fixing the values of λ_1, λ_3, λ_4, and λ_6. We determine the dynamic range, potency, sensitivity, and the amount of free signaling protein (E) at the EC_{50} for each resulting biosensor response curve (as described above) and repeat the calculation for different values of the non-dimensional parameters to generate the heat maps (Fig. S7). In the case of the intramolecular biosensor (Fig. S7A-D) we fix $\lambda_4 \gg 1$ while varying λ_1 and λ_3. In the case of the intermolecular biosensor (Fig. S7E-H) we fix $\lambda_3 \gg 1$ while varying λ_1 and λ_4. In both cases we fix $\lambda_6 = 0.01$ to allow enzyme saturation and ultrasensitivity; if $\lambda_6 = 1$ then ultrasensitivity is never observed. Using these heat maps we determine the high fidelity reporting region (Fig. 4).

Approximate analytical solution

Under quasi-steady state assumption, kinase activity (v_E) and phosphatase activity (v_F) can be described using Michaelis-Menten kinetics,

$$v_E = \tfrac{dB^*}{dt} = \frac{k^e_{\text{cat}} E B}{K^e_M + B} \tag{1}$$

$$v_F = \tfrac{dB}{dt} = \frac{k^f_{\text{cat}} F B^*}{K^f_M + B^*} \tag{2}$$

where, K^e_M and K^f_M are Michaelis-constants for the kinase and phosphatase, respectively. Allowing intermolecular and intramolecular biosensor interactions and limiting ourselves to the case where maximum oligomer is a dimer, we have the following conservation equation for the total biosensor (B_T),

$$B_T = B + B^* + B^*_c + 2B_2 + 2B^*_2 \tag{3}$$

Assuming that intermolecular and intramolecular reactions take place faster than enzymatic reactions, we can use a fast equilibrium approximation to obtain expressions for B_c^*, B_2 and B_2^* in terms of free B and B^*. Substituting these in the equation for B_T, we have,

$$B_T = B + B^* + B^*/K_D^1 + 2BB^*/K_D^2 + 2B^*B^*/K_D^2 \tag{4}$$

where, K_D^1 and K_D^2 are the dissociation constants of the intramolecular and intermolecular interactions, respectively. By solving for B in Eqn. 4, we obtain,

$$B = \frac{B_T - (1 + 1/K_D^1)B^* - 2B^{*2}/K_D^2}{1 + 2B^*/K_D^2} \tag{5}$$

We can substitute B as a function of B^* in Eqn. 1 above. The kinase and phosphtase activity then can be plotted as a function of B^* and their intersection represent the equilibrium value of B^*. An analytical expression for B^* as a root of the cubic equation can be obtained by equating Eqn. 1 and 2 or alternatively, the equilibrium B^* can be obtained numerically as we have done in Fig. S5.

This analysis is also helpful to shed light on the dependence of EC_{50} to model parameters. At $E = EC_{50}$, we denote concentration of B and B^* by B_{EC50} and B^*_{EC50}. By equating v_E and v_F from Eqn. 1 and 2 at EC_{50} we obtain:

$$EC_{50} = F \, \frac{k^f_{\text{cat}}}{k^e_{\text{cat}}} \, \frac{B^*_{EC50}}{B_{EC50}} \, \frac{K^e_M + B_{EC50}}{K^f_M + B^*_{EC50}} \tag{6}$$

In the presence of intermolecular or intramolecular interactions and due to sequestration of free biosensor maximum B^* is smaller than B_T (can be seen from Eqn. 4 for $B = 0$) while the maximum free B is still B_T. Similarly, it can be seen that at $E = EC_{50}$, we also have $B^*_{EC50} < B_{EC50}$. As can be seen from Eqn. 6 this results in always a reduction in EC_{50}. Also, if K_Ms are small this effect become weaker as EC_50 becomes independent of B_{EC50} and B^*_{EC50}. These match the results shown in Fig. S7.

This analysis shows that the mechanism of reduced ultrasensitivity is substrate sequestration. For the sequestration to work, we have assumed throughout this paper that B and B^* cannot be modified while in the B_c^*, B_2 and B_2^* states (or in higher order oligomeric states in the general model). Assuming that B^* cannot be dephosphorylated in B_2 is critical to our results but we can relax this assumption about B within the B_2 complex. In this case, we need to replace Eqn. 1 by

$$v_E \quad = \frac{dB^*}{dt} \quad = \frac{k^e_{\text{cat}} E(B + B^2)}{K^e_M + B + B^2} \tag{7}$$

So, we can solve for $B + B^2$ from Eqn. 4 and substitute it into Eqn. 7 and repeat the analysis in Fig. S5. This analysis suggests that assuming that B cannot be modified while in the B^2 state contributes to the decrease in ultrasensitivity and contributes to maintaining a constant EC_{50} but does not change our qualitative conclusions (not shown).

BioNetGen code used to generate mathematical model

```
#Model of a kinase (E) and phosphatase (F) actin on a biosensor (B).

begin parameters

#Modification module

#Enzymatic reaction (kinase)
Ekf     10
Ekb     1
Ekc     1

#Enzymatic reaction (phosphatase)
Fkf     10
Fkb     1
Fkc     1

#Intramolecular module
kon1    1000
koff1   1

#Intermolecular module
kon2    10
koff2   1

end parameters

begin molecule types
E(b)
F(b)
B(e~0~1,b,Y~U~P)
end  molecule types

begin seed species
E(b)            1
F(b)            1
B(e~0,b,Y~U)    100
end seed species

begin reaction rules

#Phosphorylation by E.
E(b) + B(e~0,b,Y~U) <-> E(b!1).B(e~1,b,Y~U!1)  Ekf,Ekb
E(b!1).B(e~1,b,Y~U!1) -> E(b) + B(e~0,b,Y~P)   Ekc

#Dephosphorylation by F.
F(b) + B(e~0,b,Y~P) <-> F(b!1).B(e~1,b,Y~P!1)  Fkf,Fkb
F(b!1).B(e~1,b,Y~P!1) -> F(b) + B(e~0,b,Y~U)   Fkc

#Intramolecular reaction.
B(e~0,b,Y~P) <-> B(e~0,b!1,Y~P!1)   kon1,koff1

#Intermolecular reaction.
```

```
B(e~0,Y~P) + B(e~0,b) <-> B(e~0,Y~P!1).B(e~0,b!1)    kon2,koff2

end reaction rules

begin observables
#Biosensor in State 1
    Molecules W B(e~0,b,Y~P),B(e~0,b,Y~U),B(e~1,b)
#Biosensor in State 2
    Molecules U B(e~0,b!1,Y~P!1)
#Biosensor in State 3 (combination of all states below, see manuscript for
   details)
    Species   V2 B==2
    Species   V3 B==3
    Species   V4 B==4
    Species   V5 B==5
    Species   V6 B==6
    Species   V7 B==7
    Species   V8 B==8
    Species   V9 B==9
    Species   V10 B==10
    Species   V11 B==11
    Species   V12 B==12
    Species   V13 B==13
    Species   V14 B==14
    Species   V15 B==15
#Sequestered signaling protein
    Molecules  E E(b!+)
end observables

begin actions

#Generate network with a maximum oligomer of size 15
generate_network({overwrite=>1,max_agg=>15});

#Generate network with a maximum oligomer of size 2
generate_network({overwrite=>1,max_agg=>2});

#Generate Matlab file
writeMfile({});

end actions
```

Supporting Figures

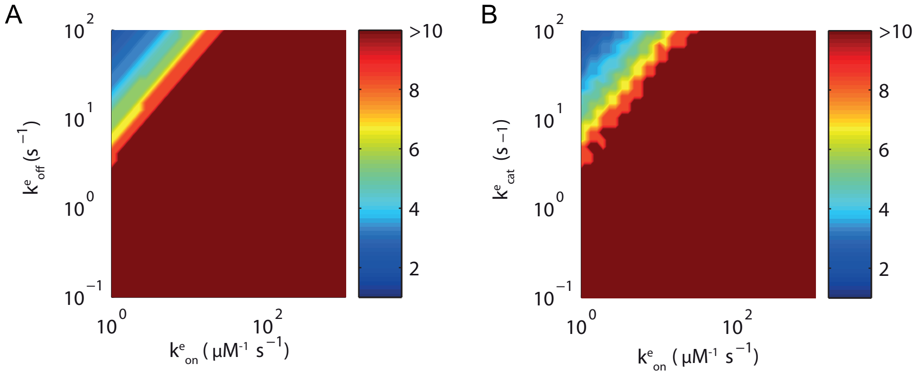

Fig S1: Effects of modifying the kinase-biosensor binding and reaction parameters on the Hill number for the biosensor architecture in Fig. 2. Heat maps of the Hill number determined from biosensor response curves as a function of the kinase-biosensor A) on-rate and off-rate and B) on-rate and catalytic-rate. Reductions in Hill numbers are observed when the off-rate or catalytic-rate are large and when the on-rate is small because in this regime, the Michaelis-constant (K_M) is large. Parameters: [Biosensor]= $B_{\mathrm{T}} = 100$ μM, $F_{\mathrm{T}} = 1$ μM, $k^1_{\mathrm{on}} = 10$ s^{-1}, $k^1_{\mathrm{off}} = 1$ s^{-1}, $k^e_{\mathrm{on}} = k^f_{\mathrm{on}}$ =10 μM^{-1}s^{-1}, $k^e_{\mathrm{off}} = k^e_{\mathrm{off}} = 1$ s^{-1}, $k^e_{\mathrm{cat}} = k^f_{\mathrm{cat}} = 1$ s^{-1}.

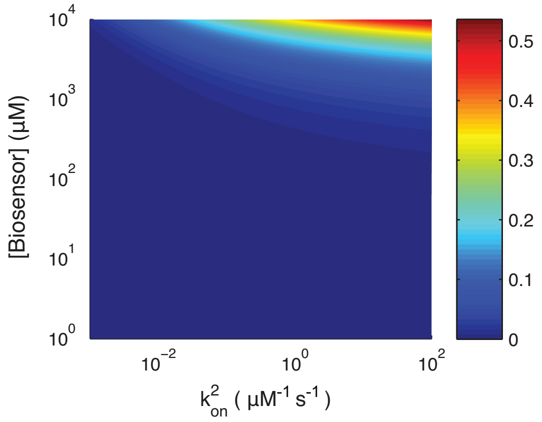

Fig S2: Heat map showing the fraction of biosensor in an oligomer of size 15 (maximum oligomer size) as a function of the intermolecular on-rate (x-axis) and the biosensor concentration (y-axis) for the parameters used to generate Fig. 3. The fraction is found to be less than 1% over the majority of parameter space indicating that approximating the maximum number of biosensors in an oligomer to 15 does not introduce appreciable errors.

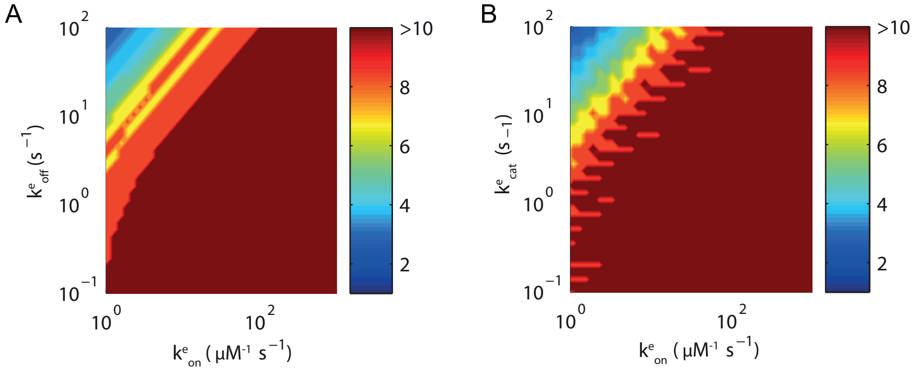

Fig S3: Effects of modifying the kinase-biosensor binding and reaction parameters on the Hill number for the biosensor architecture in Fig. 3. Heat maps of the Hill number determined from biosensor response curves as a function of the kinase-biosensor A) on-rate and off-rate and B) on-rate and catalytic-rate. As in Fig. S1, reductions in Hill numbers are observed when the Michaelis-constant (K_M) is large. Parameters: [Biosensor]= $B_{\mathrm{T}} = 100$ μM, $F_{\mathrm{T}} = 1$ μM, $k^2_{\mathrm{on}} = 0.1$ $\mu\mathrm{M}^{-1}\mathrm{s}^{-1}$, $k^2_{\mathrm{off}} = 1$ s^{-1}, $k^e_{\mathrm{on}} = k^f_{\mathrm{on}}$ =10 $\mu\mathrm{M}^{-1}\mathrm{s}^{-1}$, $k^e_{\mathrm{off}} = k^e_{\mathrm{off}} = 1$ s^{-1}, $k^e_{\mathrm{cat}} = k^f_{\mathrm{cat}} = 1$ s^{-1}.

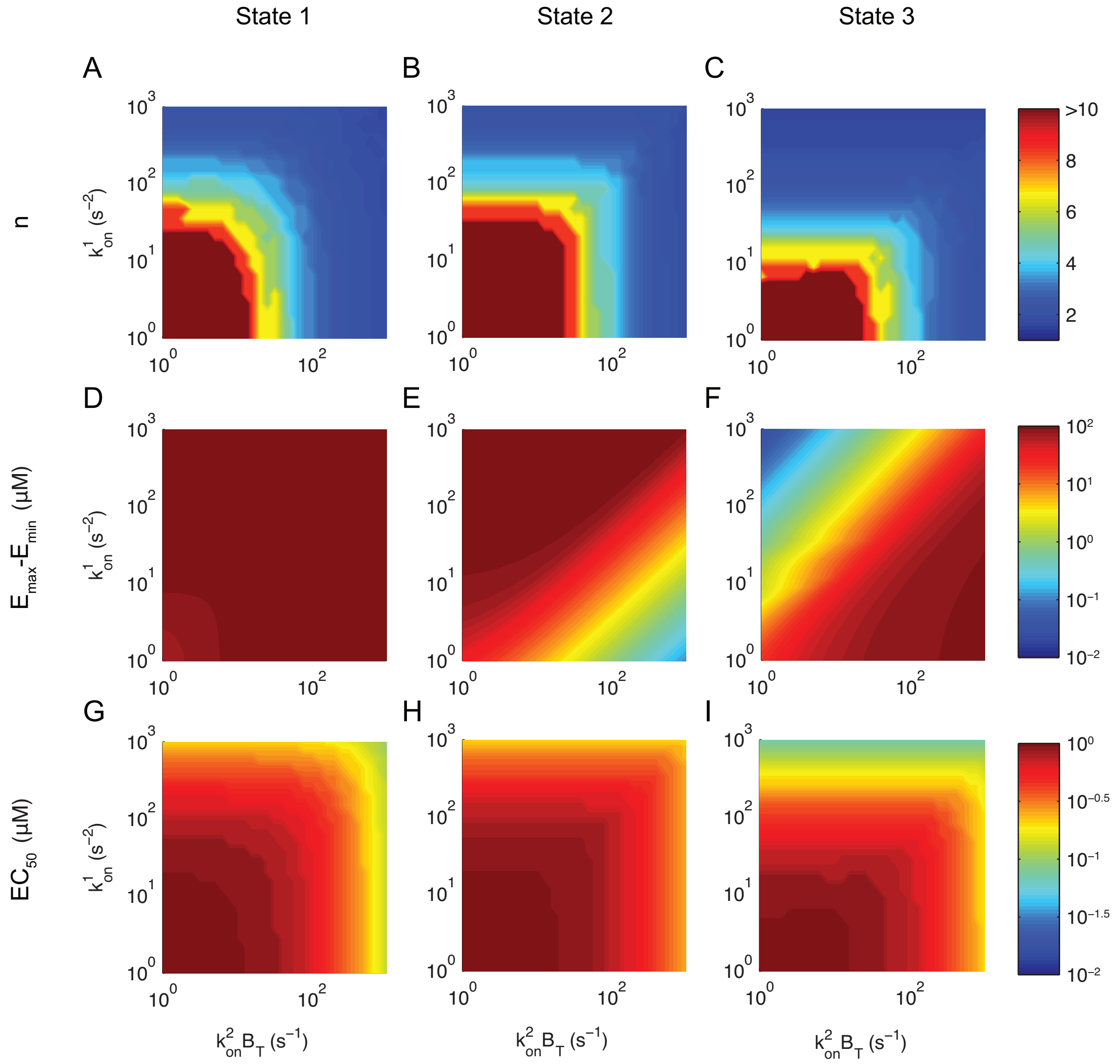

Fig S4: Biosensors exhibiting both intramolecular and intermolecular reactions do not exhibit cooperativity and introduce complex FRET states. When both intramolecular and intermolecular reactions are appreciable, at least three distinct FRET states are possible: a state where the binding domain and the binding motif are both free (State 1), 2) a state when the binding domain is intramolecularly bound to the binding motif (State 2), or 3) a state where the binding domain and/or binding motif is bound to another biosensor, possibly generating large oligomers (State 3). Heat maps showing the Hill number (n) for A) State 1, B) State 2, and C) State 3 as a function of the intramolecular (y-axis) and intermolecular (x-axis) binding rate constants. Note that when, for example, k^1_{on} is large and k^2_{on} is small the concentration of biosensor in oligomers is small. By examining State 1, which is always present, we find that there is no overt cooperativity between intramolecular and intermolecular reactions in reducing the Hill number. Heat maps for D-F) dynamic range and G-I) potency are also shown. Default parameters: [Biosensor]= $B_{\text{T}} = 100\ \mu$M, $F_{\text{T}} = 1\ \mu$M, $k^1_{\text{off}} = 1$ s^{-1}, $k^2_{\text{off}} = 1$ s^{-1}, $k^e_{\text{on}} = k^f_{\text{on}} = 10\ \mu\text{M}^{-1}\text{s}^{-1}$, $k^e_{\text{off}} = k^e_{\text{off}} = 1$ s^{-1}, $k^e_{\text{cat}} = k^f_{\text{cat}} = 1$ s^{-1}.

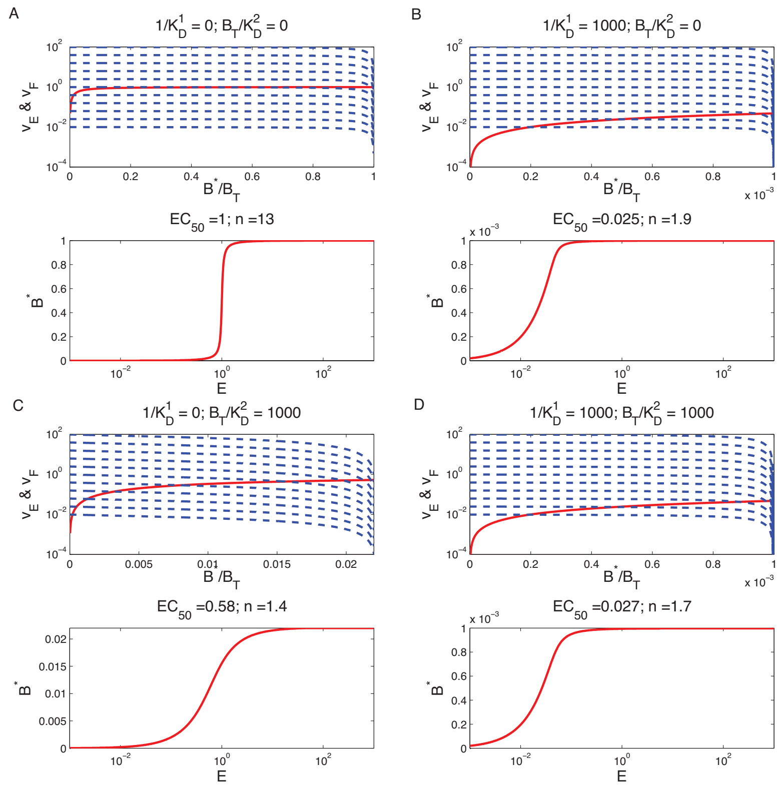

Fig S5: Intramolecular and intermolecular interactions sequester free biosensor, reducing enzyme saturation and ultrasensitivity. Kinase (dashed blue lines) and phosphatase (red solid line) activity as a function of B^*/B_T based on the approximative analytical solution (see text in Supporting Materials) for A) no intramolecular and intermolecular interaction, B) only intramolecular interaction, C) only intermolecular interaction, and D) both intramolecular and intermolecular interactions. The intersection of the kinase and phosphatase activity curves for different kinase levels determines the equilibrium value of B^*, which is plotted as a function of the kinase concentration in the lower part of each panel. The Hill number (n) and potency (EC_{50}) are also indicated. The intramolecular interaction reduces the maximum B^* (compared x-axis scales) by sequestering biosensors in B_c^*, which reduces the ability of the phosphatase to operate in the zero-order regime and hence shifts EC_{50}. Intermolecular interactions are less effective in reducing maximum B^* (compare x-axis scales), but nevertheless is able to reduce the ability of both enzymes to operate in the zero-order regime which reduces ultrasensitivity while only introducing small changes in EC_{50}. No cooperativity between intramolecular and intermolecular interactions is observed in reducing Hill numbers. Parameters: [Biosensor]= B_T = 10 μM, F_T = 1 μM, k_on^1 = 0 s^{-1}, k_off^1 = 1 s^{-1}, k_on^2 = 10 μM^{-1}s^{-1}, k_off^2 = 1 s^{-1}, $k_\mathrm{on}^e = k_\mathrm{on}^f$ =10 μM^{-1}s^{-1}, $k_\mathrm{off}^e = k_\mathrm{off}^e$ = 1 s^{-1}, $k_\mathrm{cat}^e = k_\mathrm{cat}^f$ = 1 s^{-1}.

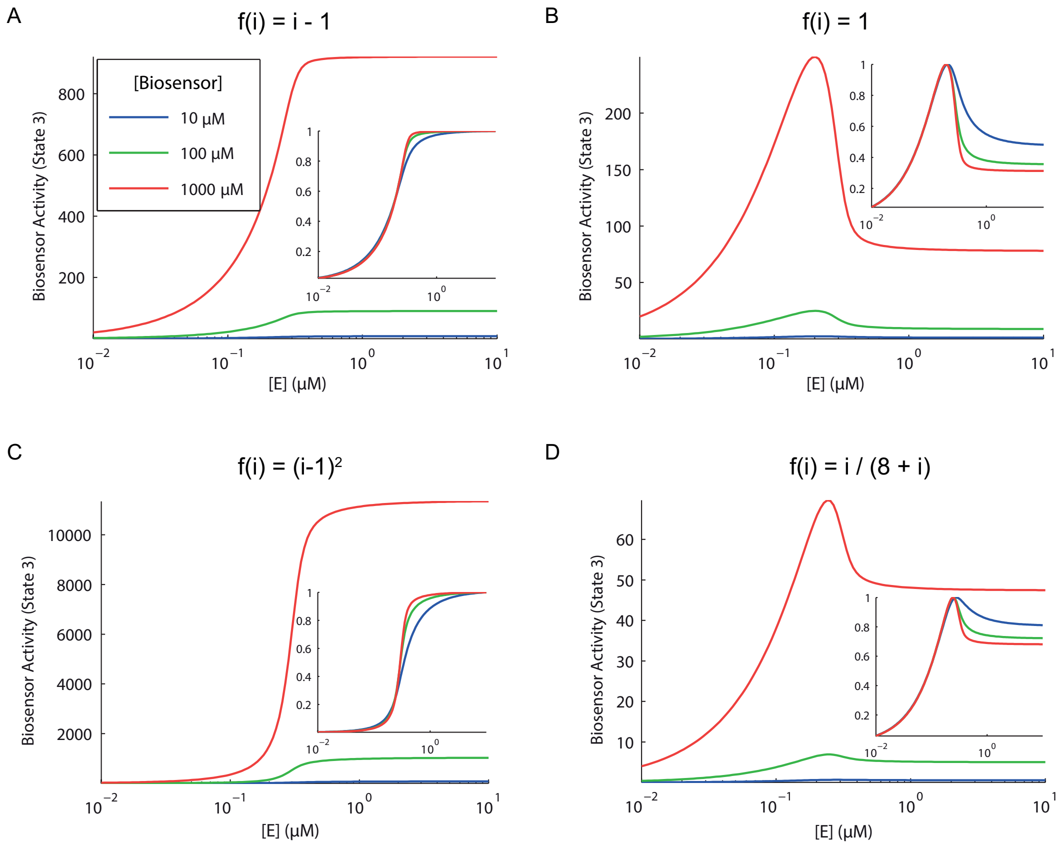

Fig S6: Biosensor activity strongly depends on the FRET kernel. The biosensor activity arising from a biosensor oligomer will depend on the oligomer size. Therefore we compute the biosensor activity as $\sum_{i=2}^{N} f(i)(B_i^* + B_i)$ where $f(i)$ is the FRET kernel. B_i^* and B_i are the concentration of biosensor in an oligomer of size i whose free tyrosine is phosphorylated or unphosphorylated, respectively. A-D) The biosensor activity is shown for the indicated FRET kernel but with otherwise identical parameters. It is evident that the FRET kernel can have a dramatic impact on the relationship between the enzyme and biosensor activity. Parameters: $F_\mathrm{T} = 1\ \mu$M, $k_\mathrm{on}^1 = 0\ \mathrm{s}^{-1}$, $k_\mathrm{off}^1 = 1\ \mathrm{s}^{-1}$, $k_\mathrm{on}^2 = 10\ \mu\mathrm{M}^{-1}\mathrm{s}^{-1}$, $k_\mathrm{off}^2 = 1\ \mathrm{s}^{-1}$, $k_\mathrm{on}^e = k_\mathrm{on}^f = 10\ \mu\mathrm{M}^{-1}\mathrm{s}^{-1}$, $k_\mathrm{off}^e = k_\mathrm{off}^e = 1\ \mathrm{s}^{-1}$, $k_\mathrm{cat}^e = k_\mathrm{cat}^f = 1\ \mathrm{s}^{-1}$.

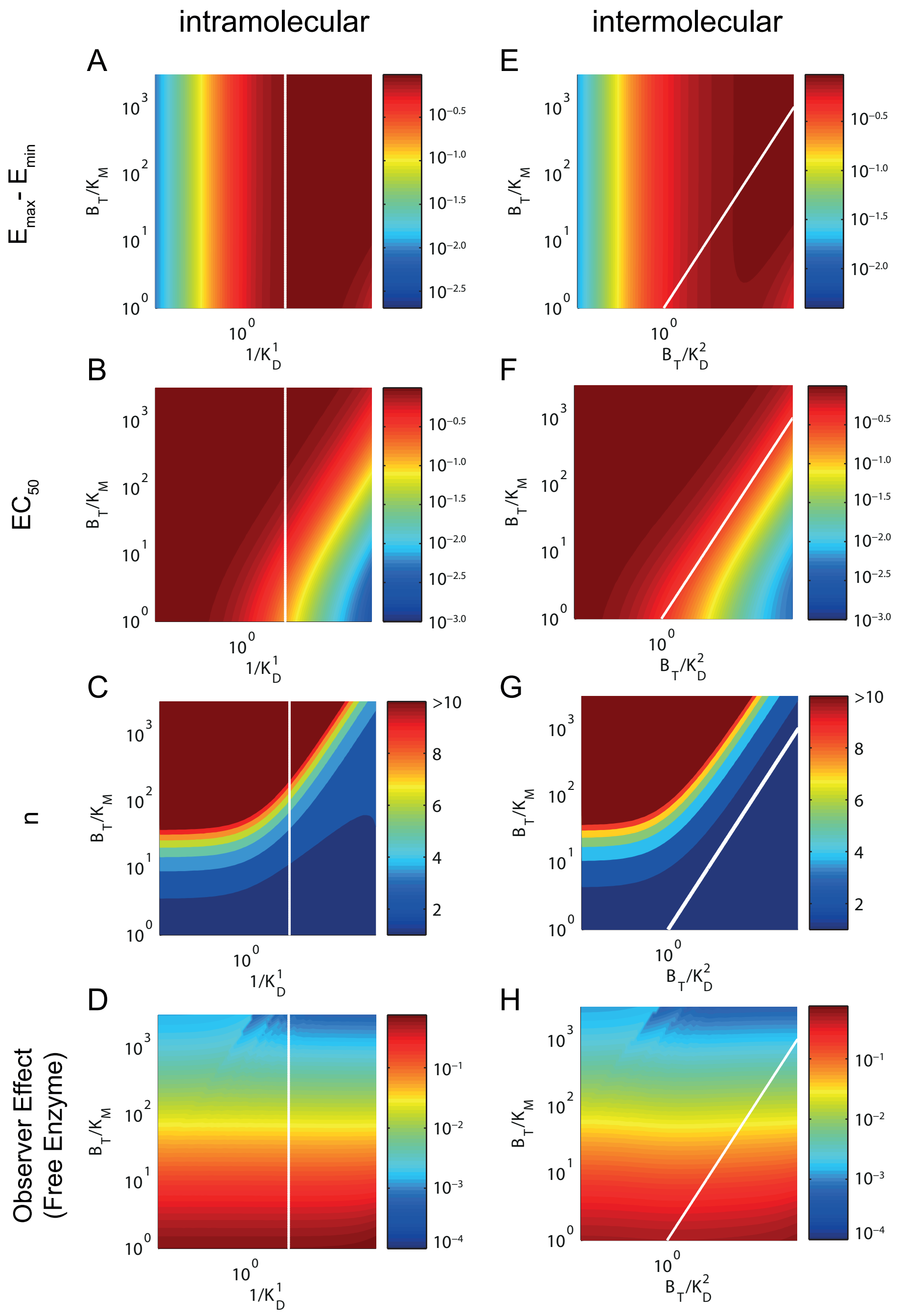

Fig S7: Non-dimensional phase diagrams of biosensor response curve measures. Results are shown for an A-D) exclusively intramolecular biosensor and E-H) exclusively intermolecular biosensor where the maximum biosensor oligomer is of size 2 (dimer). The dynamic range (A,E), potency (B,F), sensitivity (C,G), and observer effect (D,H) are as defined in the main text. White lines illustrate changes in the biosensor concentration (A-D: $K_M = 0.1$ μM and $K_D^1 = 0.1$, E-H) $K_M = K_D^2 = 0.1$ μM). Note that all heat map values are derived directly from non-dimensional calculations (e.g. EC_{50} is the ratio of E_T/F_T at half maximal dynamic range, $(E_{max} - E_{min})/2$, where E_{max} and E_{min} are the maximum and minimum fractions of active biosensor).
